# Tropical forest loss and geographic location drive the functional genomic diversity of an endangered palm tree

**DOI:** 10.1111/eva.13525

**Published:** 2023-06-15

**Authors:** Alesandro Souza Santos, Eliana Cazetta, Deborah Faria, Thâmara Moura Lima, Maria Teresa Gomes Lopes, Carolina da Silva Carvalho, Alessandro Alves‐Pereira, José Carlos Morante‐Filho, Fernanda Amato Gaiotto

**Affiliations:** ^1^ Laboratório de Ecologia Aplicada à Conservação, Programa de Pós‐Graduação em Ecologia e Conservação da Biodiversidade Universidade Estadual de Santa Cruz Ilhéus Brazil; ^2^ Laboratório de Marcadores Moleculares, Centro de Biotecnologia e Genética Universidade Estadual de Santa Cruz Ilhéus Brazil; ^3^ Instituto Federal de Educação, Ciência e Tecnologia da Bahia – Campus Seabra Seabra Brazil; ^4^ Universidade Federal do Amazonas Manaus Brazil; ^5^ Instituto Tecnológico Vale Belém Brazil; ^6^ Departamento de Biologia Vegetal Universidade Estadual de Campinas Campinas Brazil

**Keywords:** conservation genetic, local adaptation, molecular ecology, population genomics, rainforest

## Abstract

Human activity has diminished forests in different terrestrial ecosystems. This is well illustrated in the Brazilian Atlantic Forest, which still hosts high levels of species richness and endemism, even with only 28% of its original extent remaining. The consequences of such forest loss in remaining populations can be investigated with several approaches, including the genomic perspective, which allows a broader understanding of how human disturbance influences the genetic variability in natural populations. In this context, our study investigated the genomic responses of *Euterpe edulis* Martius, an endangered palm tree, in forest remnants located in landscapes presenting different forest cover amount and composed by distinct bird assemblage that disperse its seeds. We sampled 22 areas of the Brazilian Atlantic Forest in four regions using SNP markers inserted into transcribed regions of the genome of *E. edulis*, distinguishing neutral loci from those putatively under natural selection (outlier). We demonstrate that populations show patterns of structure and genetic variability that differ between regions, as a possible reflection of deforestation and biogeographic histories. Deforested landscapes still maintain high neutral genetic diversity due to gene flow over short distances. Overall, we not only support previous evidence with microsatellite markers, but also show that deforestation can influence the genetic variability outlier, in the scenario of selective pressures imposed by these stressful environments. Based on our findings, we suggest that, to protect genetic diversity in the long term, it is necessary to reforest and enrich deforested areas, using seeds from populations in the same management target region.

## 1 INTRODUCTION

To meet the demands of an ever‐increasing population, humans have drastically altered the environment, converting natural landscapes into human‐modified landscapes (Curtis et al., 2018). These anthropogenic actions cause a rapid decrease and fragmentation of native forests, leading to changes in the natural dynamics of these ecosystems and posing a major threat to biodiversity (Arroyo‐Rodríguez et al., 2020; Curtis et al., 2018). In particular, habitat loss has affected tropical forests, threatening their rich associated biota (Arroyo‐Rodríguez et al., 2020; Benchimol, Mariano‐Neto, et al., 2017; Benchimol, Talora, et al., 2017; Morante‐Filho et al., 2015; Rocha‐Santos et al., 2016). Several studies have reported that this disturbance in tropical forests cause the loss of species (Lima & Mariano‐Neto, 2014), ecological processes (Moran & Catterall, 2014) and genetic variability (González et al., 2020).

The assessment of the genetic variability in natural populations is extremely important from ecological, evolutionary and conservation perspectives in the face of constant anthropogenic disturbances (Brancalion et al., 2018; Carvalho et al., 2017; Lanes et al., 2018; Santos et al., 2015), since this variability is the raw material on which natural selection can act, allowing species to adapt to these disturbances over time (Orr, 2005; Wright, 1931). Genetic investigations using genomic approaches allow a broader understanding of how such disturbances influence the genetic variability in populations in different landscapes. Besides, they are essential for proposing conservation strategies (Ćalić et al., 2016; Fischer et al., 2013; Rellstab et al., 2015; Schoville et al., 2012). These approaches have great inferential power, because they are based on the evaluation of thousands of sites in the genome at once in a target species, while other techniques use only a limited number of markers (Bragg et al., 2015; Ćalić et al., 2016; Manel et al., 2010). Genomics also can focus on genetic variation that is either neutral (i.e., it does not influence the individual fitness) or adaptive (i.e., it does influence the individual fitness) (Holderegger et al., 2010; Manel et al., 2010). In the first case, studies are mainly interested in gene flow and genetic drift in landscapes (Holderegger et al., 2010; Storfer et al., 2010). On the other hand, the studies of adaptive genetic variation associate loci under selection (i.e., outlier loci) with environmental variables (Ahrens et al., 2018; Parisod & Holderegger, 2012; Steane et al., 2014), for example, describing genetic variation as a response to specific environmental features. Therefore, population genomics may contribute to the understanding of the genetic adaptation and species conservation in a rapid changing scenario induced by human activities (Benestan et al., 2016; Fitzpatrick & Keller, 2015; Forester et al., 2016; Sork, 2018).

In the context of tropical forests, population genomics approaches are especially relevant for the Brazilian Atlantic Forest. This forest domain has high species richness and endemism (Martini et al., 2007; Myers et al., 2000; Thomas et al., 1998) but is currently reduced to less than 28% of its original extent, with highly fragmented and disturbed remaining forests (Rezende et al., 2018). Therefore, understanding the consequences of anthropic disturbances on the genomic variability in species found in this biome is important to design and implement efficient forest conservation strategies (Santos & Gaiotto, 2020).

We selected the native palm *Euterpe edulis* Martius (Arecaceae) as a biological model to understand the effects of landscape‐scale forest loss and fragmentation on the genomic variability. This species occurs throughout the Brazilian Atlantic Forest and has great ecological importance since its fruits are consumed by approximately 58 species of birds and 20 species of mammals. It has being considered a key species for maintaining fauna in periods of resource scarcity (Galetti et al., 2013). In economic terms, *E. edulis* has one of the most widely used (i.e., overexploited) nontimber products in the Brazilian Atlantic Forest. Its apical meristem (heart of palm), usually obtained by illegal extraction, is very desired for human consumption (Brancalion et al., 2012; Muler et al., 2014). Heart of palm extraction causes death to adult individuals and, consequently, a reduction in their populations, which together with the loss and fragmentation of their habitats, has included *E. edulis* on the endangered Brazilian flora list (Martinelli & Moraes, 2013). In fact, demographic studies demonstrate local extinction of this species in severely deforested landscapes (Leal et al., 2021) and low growth and survival potential of young individuals in deforested landscapes under high light penetration (Cerqueira et al., 2021). From a molecular point of view, studies using neutral microsatellite markers (i.e., with no influence on individual fitness) have shown that the loss and fragmentation of forests can lead to the collapse of gene flow between populations and increase the inbreeding coefficient (Carvalho et al., 2016, 2017; Pereira et al., 2022; Santos et al., 2016). Even located in selectively neutral locations, these markers are considered representative of the whole genome. Therefore, they indicate what is happening with the population in terms of genetics. In addition, forest loss can have indirect effects on this species, reducing the richness and/or abundance of birds that disperse its seeds leading to microevolutionary and phenotypic changes (Carvalho et al., 2016; Galetti et al., 2013). Synergistically, this dispersers loss can reduce the genetic variability in remaining populations limiting gene flow by seeds (Santos et al., 2016). In addition, populations inserted in different biogeographic regions, such as semideciduous forest vegetation and rainforest, have different gene pools, due to the distinct selective regimes imposed by regions (Carvalho et al., 2016).

In the present study, we aimed to investigate the patterns of variability and genetic structure in *E. edulis* populations, using SNP markers inserted in transcribed regions of genome, which may help to understand the local adaptation of this important species. Using this approach, we predicted that (i) genetic structure and variability of populations reflect regional characteristics, due to the different selection regimes imposed by the biogeographic regions and/or different deforestation histories; (ii) landscape forest loss as well as reduction in the richness and abundance of seed‐dispersing birds reduce the neutral genetic variability in populations due to genetic drift and a decrease in gene flow; and (iii) changes in environmental or ecological conditions (e.g., a reduction in the richness and abundance of seed dispersers or loss of forest) may impose selective pressures, favoring the detection of genes under selection (i.e., outliers) in *E. edulis* populations.

## 2 MATERIALS AND METHODS

### 2.1 Study area

**FIGURE 1:**
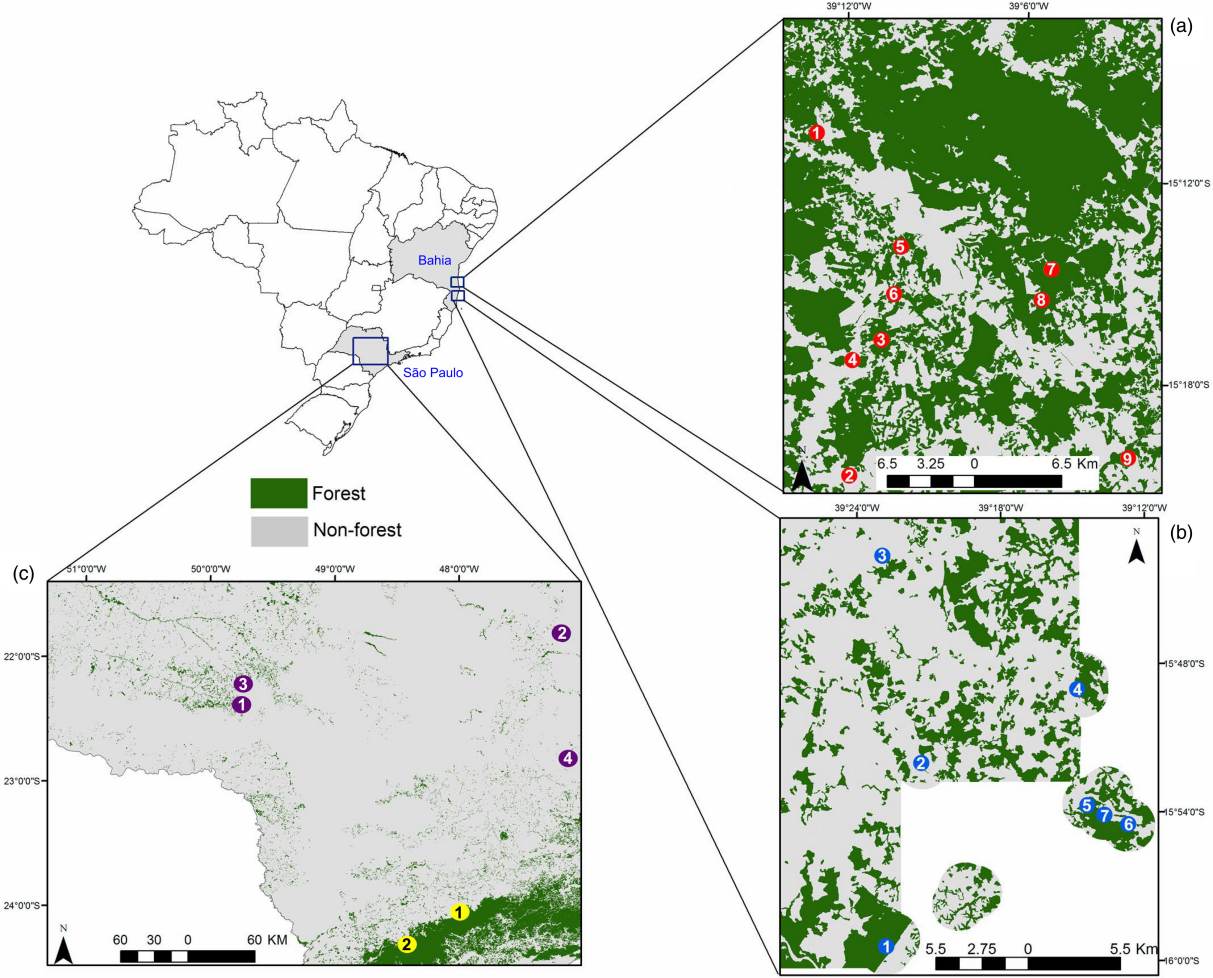
Geographic distribution of the 22 populations of *Euterpe edulis* sampled in fragments of the Atlantic Forest located in the states of Bahia and São Paulo. (a) Southern Bahia, northern region (BA_NR region; red sites); (b) southern Bahia, southern region (BA_SR region; blue sites; (c) São Paulo, rainforest (SP_RA region; yellow sites) and semideciduous forest (SP_SE region; purple sites). The numbers correspond to the identification of each population within their respective region (see Table S1 for more details).

The study was performed in remnants at the Atlantic Forest located in the states of Bahia (BA) and São Paulo (SP), Brazil (Figure 1).

In Bahia, we sampled in 16 forest fragments located in the southern of this state, comprising one of the largest remnants of the Atlantic Forest in northeastern Brazil (Rezende et al., 2018; Thomas et al., 1998). This area is considered a high priority for conservation due to the high richness and endemism, mainly of tree species (Martini et al., 2007). These 16 fragments are distributed in two regions with similarities in flora, topography, and soil type (Thomas et al., 1998), but differing in the amount of remaining forest cover (Figure 1; for more details, see Morante‐Filho et al., 2016). In the first region (BA_NR), 50% of the native forest remains, comprising large and continuous forest fragments, such as the Biological Reserve and the Una Wildlife Refuge (together representing 34.804 ha of protected area), and a matrix dominated by cocoa agroforestry. The second region (BA_SR), contrasted drastically with BA_NR, with only 30% of the native forest remaining and consisting of a matrix dominated by open areas (Morante‐Filho et al., 2016). So, although the deforestation process in Bahia started in the mid‐1980s but was intensified in the 1990s (Rocha, 2006), deforestation started in BA_SR and then moved toward BA_NR, that is, in BA_SR deforestation is older.

The Atlantic Forest in SP has a history of deforestation for planting monocultures, such as coffee, that dates back to the 19th century (Dean, 1996). In this state, we sampled six fragments of Atlantic Forest characterized by semideciduous forest vegetation (SP_SE region) and rainforest (SP_RA region). These regions differ in the plant species composition as well as in the prevailing environmental conditions (Eisenlohr & de Oliveira‐Filho, 2015). For example, while the SP_SE region is in the interior of the state and has a sharp dry season and nutrient‐rich soils, the SP_RA region is at the coast, does not experience rainfall limitations throughout the year, and has poor nutrient soils (Eisenlohr & de Oliveira‐Filho, 2015).

### 2.2 Sampling design

For the southern region of BA, we analyzed a land use map based on high‐resolution satellite images (Quick Bird and World View, both from 2011, and Rapid Eye from 2009 to 2010) that covered 3470 km^2^ at a scale of 1: 10,000. We identified 58 potential sampling sites located in landscapes that showed great variation in the amount of remaining forest. Then, we selected 16 forest sites with a minimum distance of 1 km from each other (Table S1); nine of these sites are located in BA_NR region and other seven sites are in BA_SR region. Based on previous studies (Santos et al., 2015; Soares et al., 2019), all site present occurrence data of *E. edulis*. Based on this mapping approach, native forest cover (%) within a landscape radius of 2 km from the center of each selected forest location was calculated using the Quantum GIS 3.20.3 program (QGIS Development Team, 2021). Each landscape was defined by a radius of 2 km, considering the foraging distance of the large dispersers of *E. edulis* seeds less than 600 m and that the foraging distance of important pollinators of this palm tree presents a distance of less than 1 km (Holbrook, 2011; Zurbuchen et al., 2010). Furthermore, several genetic studies have successfully used the same landscape sizes to assess the effects of forest loss in this palm species (Carvalho et al., 2015, 2017; Santos et al., 2015, 2016).

For the areas sampled in SP we used information from previous studies to identify areas with *E. edulis* (Carvalho et al., 2015), and six sampling points were selected. There was great variation in the amount of forest at these points and a minimum distance of 1 km between the points. Subsequently, we used high‐resolution images from Google Earth version 7.3.2 through the Open Layers plugin in Quantum GIS 3.20.3 program (QGIS Development Team, 2021) to visually classify the forested and nonforested parts of the images. Thus, the percentage of forest in each area was quantified, and the same procedures were followed in the sampled locations in BA.

### 2.3 Focal species and sampling

*Euterpe edulis* is a monoecious species, with a preferentially allogamous reproductive system (i.e., fertilization occurs when pollen from one plant fertilizes the flower stigma of another plant), although it has self‐compatibility (Gaiotto et al., 2003). Its flowers are visited by several small insects, with *Meliponini* and *Euglossini* bees being the main pollinators (Reis et al., 2000; Santos et al., 2018). This palm bears annual fruiting, with seeds dispersed mainly by large frugivorous birds such as toucans and cotingas and by small birds such as thrushes (Carvalho, Garcia, et al., 2021; Galetti et al., 2013). *Euterpe edulis* seeds are recalcitrant and moisture dependent for germination, with evidence suggesting that deforested landscapes under high light penetration have low potential for growth and survival of young individuals (Cerqueira et al., 2021; De Andrade, 2001). The population density of *E. edulis* varies significantly along its distribution area, with the state of BA presenting approximately 1305 ind./ha, while in SP it can register populations with about 17,000 ind./ha (Leal et al., 2021; Reis et al., 2000), being the generation time of the species approximately 18 years (Carvalho, Lucas, & Côrtes, 2021; Franco & Silvertown, 2004; Galetti et al., 2013).

In the state of São Paulo, individuals were randomly collected within each fragment. In Bahia state, we allocated three 50 × 10 m plots, randomly located within each forest site, but maintaining a minimum interplot space distance of 50 m and of the nearest edge, for sampling *E. edulis*. In each forest site, we collected leaf material and georeferenced all the juvenile individuals of *E. edulis* with leaf insertion heights between 0.15 and 1.00 m and belonging to young class II, according to the classification of Silva et al. (2009). This ontogenetic stage was chosen because the genetic parameters of the early developmental stages better represent the effects of recent environmental disturbances (Vranckx et al., 2012). Also, in forest sites in which more than 50 individuals were collected, we randomly selected 50 individuals for genomic analysis. The number of individuals sampled per forest site was established to maximize the accuracy power of the detection of SNPs using sequencing of pooled DNA samples (Schlötterer et al., 2014). However, in eight sites, fewer than 50 individuals were found (Table S1).

### 2.4 Laboratory and bioinformatics procedures

Genomic DNA was extracted from the leaf tissues of the 1063 individuals sampled in the 22 study areas (Table S1) using the protocol described by Doyle and Doyle (1990). Equimolar amounts of DNA from individuals from the same collection area were used to form 22 pools, one for each study area. We opted for the strategy of sequencing pooled DNA samples (pool‐seq) due to its efficiency and reduced costs for obtaining SNP data in landscape genomics studies, which generally require a large number of samples (Santos & Gaiotto, 2020). This methodology has been reported as the best option for reducing costs related to next‐generation sequencing in the analysis of no‐model species (Schlötterer et al., 2014), as is the case for *E. edulis*.

To perform sequencing, libraries with barcodes were prepared for each of the 22 pooled samples, and 20,000 probes were used. These probes were previously developed to capture specific transcribed regions of the *Euterpe* genome (Sales, 2022). The DNA captured by these probes was sequenced using an Illumina HiSeq 2000 platform (Illumina). After sequencing, the 3′ end was cut to remove low‐quality bases (reading quality index < 20), and the reads were filtered to remove those with quality index values smaller than 10%. Then, the reads selected after the trimming and filtering steps were aligned using MOSAIK 2.2 (Lee et al., 2014).

In identifying the SNPs, we consider the number of samples contained in each pool, using the pooled‐discrete option of the FreeBayes 0.9.15 program (Garrison & Marth, 2012). Then, the identified SNPs were submitted to a quality filter (minQ, 10; max‐missing‐count, 3; min‐alleles, 2; max‐alleles, 2; min‐meanDP, 3; max‐meanDP, 750; maf, 0.01; and mac, 1) using the program VCFtools 0.1.12a (Danecek et al., 2011). After these two steps, 323,839 SNPs were retained. However, as there was still a large variation in the quality of mapping (QUAL) and sequencing depth (DP), we performed an additional filtering step, retaining only the markers with QUAL ≥ 100 and DP ≥ 100 (Schlötterer et al., 2014). Subsequently, only the markers with QUAL and DP between the 5th and 95th quantiles were retained (i.e., 90% retention of the markers based on the QUAL and DP distributions). Of these, we retained only the markers with the highest DPs for each of the contigs, seeking to minimize the explicit linkage disequilibrium, resulting in 8296 SNPs. However, as the computational packages consider only biallelic SNPs, a total of 7632 markers were used for genetic analysis.

### 2.5 Characterization of the global genetic structure

To assess the organization of genetic diversity and to define groups that reflect biologically plausible scenarios for conducting the tests to detect outlier markers, we performed a principal component analysis (PCA) and estimated the genetic divergence among populations based on all SNP loci. The PCA was carried out with the LEA package (Frichot & François, 2015) on the R platform (R Core Team, 2021). Genetic divergence between population pairs was estimated using the *F*_ST_ described for pools of individuals by Hivert et al. (2018), which is derived from Cockerham and Weir's (1987) *F*_ST_, using the poolfstat package (Hivert et al., 2018) on the R platform (R Core Team, 2021). The *F*_ST_ values obtained were represented on a heatmap produced with the heatmap3 package (Zhao et al., 2014) and associated with the number of groups estimated through a hierarchical clustering algorithm with the Euclidean distance and the average method. Because these analyses revealed a large genetic divergence between populations from the states of BA and SP (Figure S1A,B) and it is known that there are climatic and biological differences between them, and that the history of anthropogenic disturbances differs between states, the tests to detect outlier markers, and the other genetic analyses were applied to the populations of BA separately from the populations of SP.

### 2.6 Detection of markers potentially under selection

Among the most common tests to identify hypothetical selection signals in SNP markers (outliers) are those based on significant deviations from *F*_ST_ (or related) estimates between populations (Fariello et al., 2013). Furthermore, environmental association analyses (EAAs) can be used to identify associations between genotypes or allelic frequencies with environmental variables (Ahrens et al., 2018). In this case, the SNPs significantly associated with environmental variables can be interpreted as a subset of markers related to selective pressures (Manel et al., 2018).

Although there are several methods for identifying selection signals with SNP markers, these tests generally reveal false positives, given their different assumptions and genetic models, which do not necessarily reflect the sampling design, the genetic structure of the population in question, and other covariates (Ahrens et al., 2018; Lotterhos & Whitlock, 2015). In this context, in an attempt to minimize the impact of false positives, and taking advantage of different assumptions, two tests were used to identify outlier SNP markers (pcadapt and X^T^X statistic) and two tests of environmental association analyses (BayPass – Bayesian population association analysis and LFMM – latent factor mixed models). Based on the results of PCA and *F*_ST_ between *E. edulis* populations (Figure S1A,B), the tests were applied to the 16 populations from Bahia separately from the six populations of São Paulo.

Unlike most methods, pcadapt (Luu et al., 2017) does not assume an explicit genetic model and does not require predefined groups of individuals. It performs genomic scans through principal component analysis (PCA) and assumes that outlier markers will be related to the population structure suggested by PCA. Each marker is assigned to a *z*‐score value that quantifies it the relationship with the first K major components that best explain the observed genetic structure. Based on this *z*‐score, a Mahalanobis distance is calculated for each marker in relation to the other markers. Thus, outlier SNPs are those that present significant deviations from these distances in relation to the mean of the values of the other markers. This analysis was performed with the pcadapt package (Luu et al., 2017) on the R environment (R Core Team, 2021). For the analysis, K = 3 (Bahia) and K = 2 (São Paulo) were considered as principal components (the point at which the inclusion of more components does not cause a large increase in the cumulative proportion of variation explained by the PCA). We considered SNPs with *q*‐values ≤0.1 as outliers, assuming that 10% of outlier SNPs can be false positives.

For ByPass and LFMM analysis, we used as covariates the percentage of forest calculated in each landscape, the richness and abundance of seed dispersers (birds that defecate or regurgitate palm seeds). For the two regions in BA, we used richness and abundance data for these birds sampled in each study area (for more information, see Morante‐Filho et al., 2015), and richness data for the two regions in SP (Carvalho, Garcia, et al., 2021). The BayPass (Gautier, 2015) presents methods both for identifying outlier markers and for identifying markers hypothetically associated with specific covariates. It explicitly considers the covariance structure between allelic frequencies of populations (Ω), resulting from their shared evolutionary history. To identify outlier SNPs, the X^T^X statistic (Günther & Coop, 2013) for each locus is used. Based on simulated data using initial estimates of Ω, new estimates of X^T^X are calculated to define threshold values for identifying outlier loci. For analysis of the association between genetic and environmental variation, BayPass implements a covariation model that assesses how population covariates are associated with each marker, considering linear regression coefficients (βik), using Bayesian statistics. For the analyses using the X^T^X statistic, allelic frequency covariance estimates between populations for each locus, and regression coefficients with environmental variables were estimated from 20 pilot analyzes with 2000 iterations of MCMC, followed by a burn‐in period of 50,000 and 500,000 iterations of MCMC (Günther & Coop, 2013). After obtaining the allelic frequency covariance matrix between populations, the calibration of X^T^X values was performed with the same settings considering 5000 markers simulated with the simulate.baypass function. From this analysis, markers with X^T^X estimates above the 95% percentile of calibration estimates were considered as outlier loci. For the analysis of association between SNPs markers and environmental variables, Bayes Factor values (BF.dB) were used to compare two alternative models for each SNP (and each environmental covariate): model with association (βik ≠ 0) and model without association (βik = 0). We considered SNPs significantly associated with environmental variables those with BF.dB above the 95% percentile of the calibration estimates.

In the analysis with latent factor mixed models (LFMM), allelic frequencies are considered as response variables to environmental predictors. The LFMM considers that unobserved latent factors, such as demographic factors, are modeled as confounding effects. After that, associations between environmental variables and markers can be interpreted as hypothetical signatures of natural selection (Frichot et al., 2013). The LFMM analyses were performed in the LEA package (Frichot & François, 2015) on the R environment (R Core Team, 2021). Based on PCA and *F*_ST_ between pairs, we considered two population groups (K = 2) for each state (Figure S1A,B), and 10 repetitions of the algorithm were performed with burnin of 50,000 and 100,000 iterations. We considered SNPs significantly associated with environmental variables those with a false discovery rate (FDR) ≤ 0.05.

In this study, we considered markers putatively under selection (outlier SNPs) if they were identified as outliers by at least two of these four tests simultaneously (Figure S2A,B). In total, 1484 outlier SNPs in BA and 1053 outlier SNPs in SP were identified (Table S1), with an overlap of only 187 SNPs between BA and SP (Figure S2C). The number and overlap of the outlier SNPs identified by these methods were represented using a Venn diagram using the VennDiagram in R platform (Chen & Boutros, 2011). There was a very large variation in the number of SNPs identified by these analyses, demonstrating the importance of simultaneously using more than one method to minimize possible false positives (Ahrens et al., 2018; Lotterhos & Whitlock, 2015). Thus, ensuring greater reliability in distinguishing neutral SNPs from those putatively under natural selection allowed us to proceed with the analysis of genetic variability and statistical analysis with the two data sets separately for each state (BA and SP).

### 2.7 Genetic variability analysis

We considered as neutral SNPs those makers that were not identified as outliers by at least two of the methods described above. The estimates of the average number of neutral and outlier alleles per locus in each of the 22 populations was obtained with the adegenet package (Jombart & Ahmed, 2011), based on 4252 neutral biallelic and SNPs and 1484 outlier SNPs in BA and, 4927 neutral biallelic and SNPs and 1053 outlier SNPs in SP (Table S1). Based on the allele frequencies of the neutral and outlier loci separately, Nei's (1978) gene diversity (H_E_) was estimated and was corrected for finite population sizes (each locus and each population) according to the formula: $H_{E}=\frac{2n}{2n-1}\left(1-\sum p_{i}^{2}\right)$ where H_E_ is gene diversity, *n* is the number of individuals per population, and *p*_i_ is the frequency of the I the allele.

We also used principal component analysis (PCA) of the LEA package (Frichot & François, 2015) and *F*_ST_ of the poolfstat package (Hivert et al., 2018) to assess the structure between pairs of populations. The *F*_ST_ values obtained were represented on a heatmap produced with the heatmap3 package (Zhao et al., 2014) and associated with the number of groups estimated through a hierarchical clustering algorithm with the Euclidean distance and the average method. Then, we assessed whether geographic distance (km) influenced *F*_ST_ estimates between the populations within each state by performing a Mantel test (Mantel, 1967) using the ecodist package (Goslee & Urban, 2007). Next, to test whether the observed pattern of genetic differentiation (*F*_ST_) was due to the isolation by distance (IBD) process, we performed *F*_ST_/(1 − *F*_ST_) regression against the logarithm of geographic distances in *E. edulis* populations within each state (Rousset, 1997). All the analyses were performed in the R platform (R Core Team, 2021).

### 2.8 Statistical analysis

We performed an unbalanced ANOVA followed by a Scott‐Knott means test to assess whether the number of alleles or gene diversity of the neutral or outlier loci differed by sampled region.

For the 16 sampled populations in BA (BA_SR and BA_NR), we evaluated whether the richness and abundance of bird seed dispersers and the amount of forest cover at the landscape scale affected the number of alleles or the gene diversity of the neutral and outlier loci. We assessed only the populations from BA due to the low number of sampled populations in SP (SP_SE and SP_RA), which could bias the models. To carry out this investigation, we used data from birds that regurgitate or defecate *Euterpe* seeds (Galetti et al., 2013) in the BA_SR and BA_NR regions (For sampling details, see Morante‐Filho et al., 2015). In particular, we used three models: (1) a null model, (2) a univariate linear model, and (3) an ANCOVA model. In the linear model, we create different simple linear regression between the response variable (gene diversity or number of alleles) and the predictor variable (bird richness, abundance or forest cover). In addition, as the response variables could be influenced simultaneously by more than one predictor variable, we create models with interactions between different predictor variables. We also create several ANCOVA models, always using the study region as a categorical factor interacting with the different variables (bird richness, abundance or forest cover) and genetic parameters as the response variables. Then, we used the corrected Akaike information criterion for small samples (AIC_C_) and Akaike weights to select the most plausible model (Anderson, 2008). The model presenting the smallest AIC_C_, with at least two units of difference from those of the other models, and with the largest Akaike weights was considered the most plausible. The models were create using the bbmle package (Bolker, 2012) and plotted with ggplot2 (Wickham, 2016). All the analyses were performed in R platform (R Core Team, 2021).

## 3 RESULTS

### 3.1 Genetic diversity and structure

The 22 populations had an average number of alleles per locus of 1.696 (±0.03 SD) for neutral loci and 1.596 (±0.11 SD) for outlier loci, with an average gene diversity (H_E_) of 0.238 (±0.01 SD) for neutral loci and 0.230 (±0.05 SD) for outlier loci (Table S1).

**FIGURE 2:**
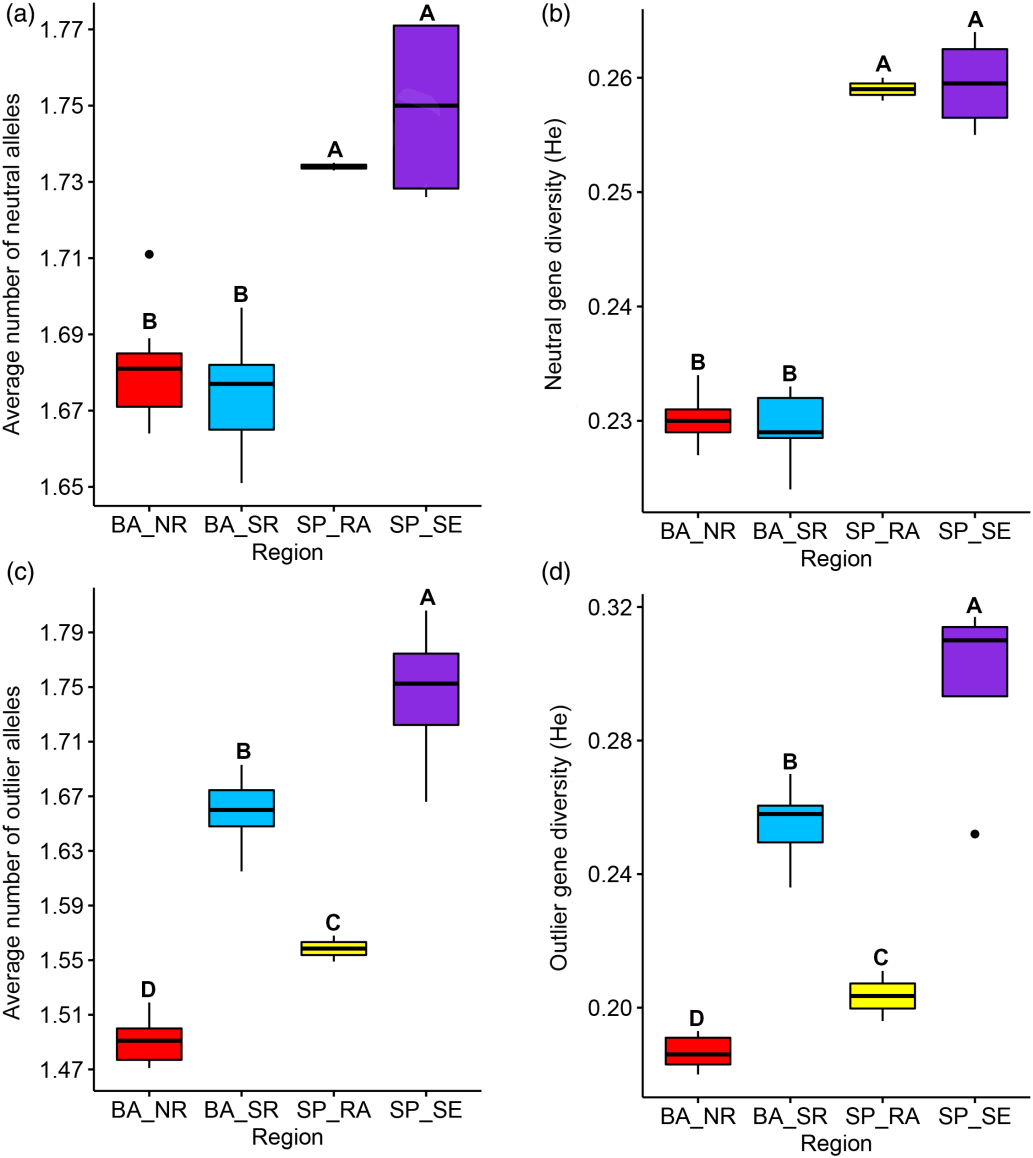
Boxplots of the Scott‐Knott averages for the estimates of genetic variability in the *Euterpe edulis* populations sampled in the four regions studied: (a) average number of alleles per neutral locus; (b) neutral gene diversity; (c) average number of alleles per outlier locus; and (d) outlier gene diversity. Different letters indicate significant differences in the average estimates among the regions.

Our results also highlight that the average number of alleles per locus and neutral gene diversity differ only between the regions of the state of Bahia (BA_NR, BA_SR) and São Paulo (SP_RA, SP_SE), with the highest averages observed in SP (Figure 2a,b, respectively). For the genetic variability estimated with the outlier SNPs, all regions differed significantly from each other, with the SP_SE region showing the highest average number of alleles per locus and gene diversity, followed by the BA_SR, SP_RA and BA_NR regions, respectively (Figure 2c,d).

**FIGURE 3:**
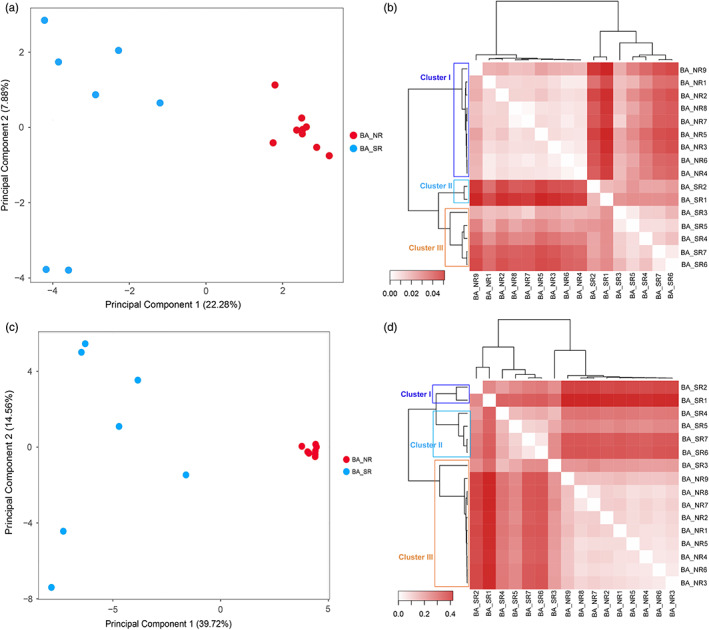
Characterization of the genetic structure with neutral (a, b) and outlier locus (c, d) of 16 populations of *Euterpe edulis* located in Atlantic Forest remnants in the Bahia state: (a, c) principal component analysis (PCA) of the populations of *E. edulis* and (b, d) heatmap based on Euclidean distance and the average clustering method using *F*_ST_ values. The three groups established (cluster I, lilac rectangle; cluster II, blue rectangle; cluster III, orange rectangle).

**FIGURE 4:**
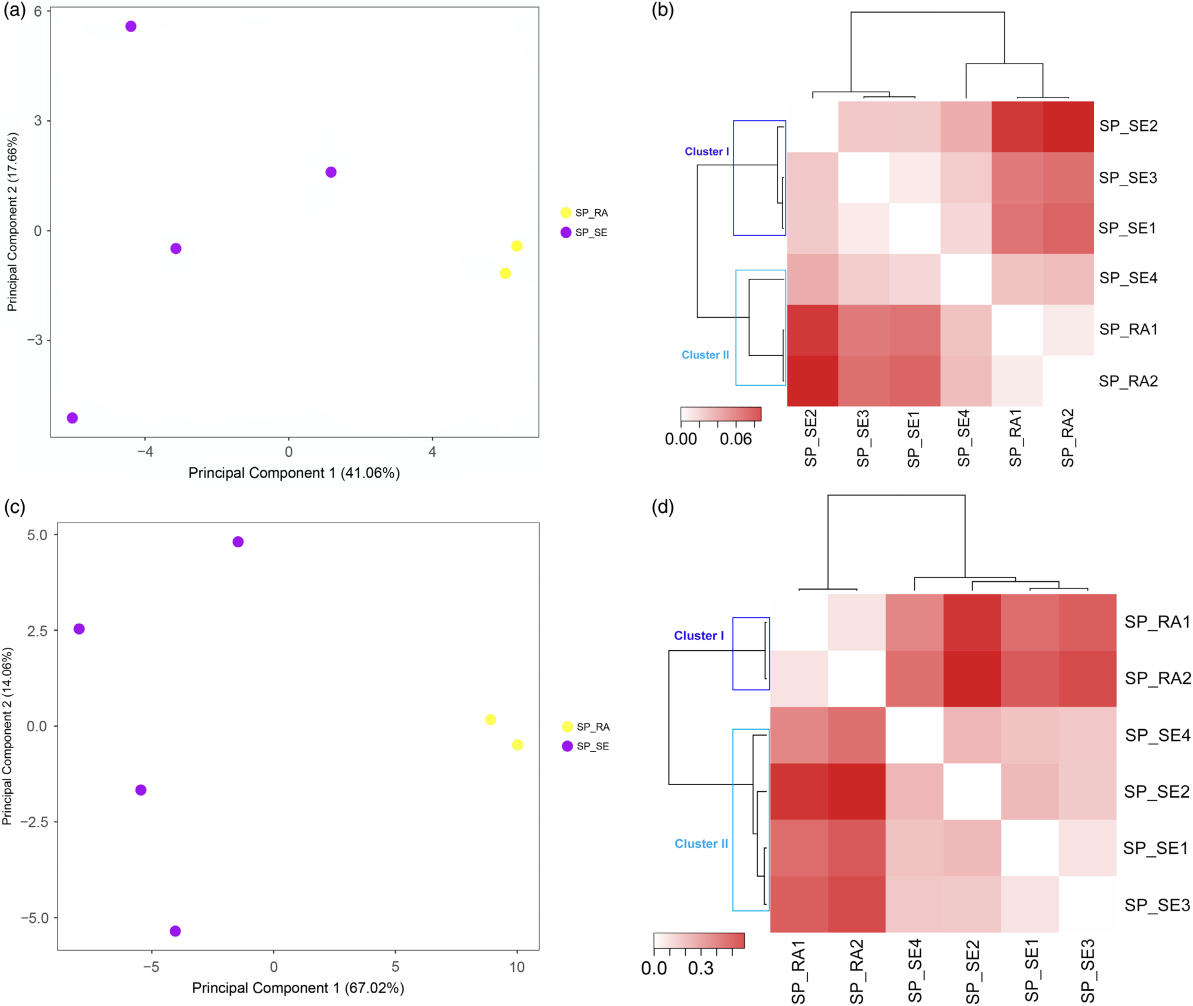
Characterization of the genetic structure with neutral (a, b) and outlier locus (c, d) of six populations of *Euterpe edulis* in São Paulo: (a, c) principal component analysis (PCA) of the populations of *E. edulis* and (b, d) heatmap based on Euclidean distance and the average clustering method using *F*_ST_ values. The two groups established (cluster I, lilac rectangle; cluster II, blue rectangle).

In the analysis of the genetic structure based on the neutral loci in BA, the first two PCA axes explained 30.16% of the variation, clearly separating the populations of BA_NR from BA_SR (Figure 3a). For the analysis of *F*_ST_ with neutral loci, we recorded the formation of three groups, separating BA_NR from the other two groups formed by BA_SR populations, just as observed in PCA (Figure 3a,b). This same pattern of regional genetic structure was also registered in the analyses with the outlier loci in BA, in which the first two PCA axes explained 54.28% of the variation (Figure 3c), and the *F*_ST_ analysis separating BA_NR from the other two groups formed by BA_SR populations (Figure 3d). For the state of SP, in the analysis of the genetic structure with neutral loci, the first two PCA axes explained 58.72% of the variation, and populations were grouped by region (Figure 4a). The *F*_ST_ analysis with neutral loci showed the formation of two groups, one with populations from the SP_SE region and the other formed by SP_RA populations with a single SP_SE population, just as observed in the PCA (Figure 4b). When considering the genetic structure with the outlier loci in SP, the first two PCA axes explained 81.08% of the variation, and populations were grouped by region, converging with the *F*_ST_ analysis (Figure 4c,d).

When evaluating the influence of geographic distance on the genetic structure (*F*_ST_) within each state with the Mantel test, significant relationships were observed for neutral (*r* = 0.84, *p* < 0.001) and outlier (*r* = 0.85, *p* < 0.001) SNPs in BA, as well as for the neutral (*r* = 0.58, *p* = 0.005) and outlier (*r* = 0.58, *p* = 0.036) SNPs in SP. However, the regression between *F*_ST_/(1 − *F*_ST_) against the log of geographic distances did not provide a linear relationship for neutral SNPs or outlier, as would be expected by the IBD process (Figure S3).

### 3.2 Influence of forest cover and avian seed dispersers on genetic variability

**FIGURE 5:**
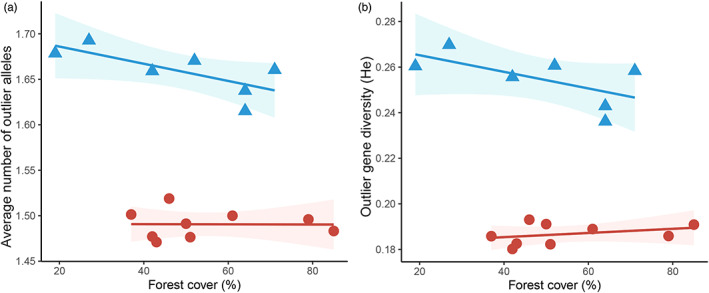
Effect of the amount of forest cover on the outlier genetic variability in *Euterpe edulis* populations located in two regions (BA_SR and BA_NR) of the Atlantic Forest in Bahia, Brazil; (a) average number of alleles per locus; (b) gene diversity. The lines represent the best fit of the most plausible model (see Table 1). In the ANCOVA model, we showed the regression lines by region, with the blue rectangles indicating the southern region (BA_SR) and the red dots indicating the northern region (BA_NR). The shaded areas correspond to the 95% confidence intervals of the models.

In the model selection process, the ANCOVA composed of the percentage of forest cover interacting with the region was the most plausible model to explain the genetic estimators assessed with the outlier SNPs. On the other hand, the null model was the most plausible to explain the genetic estimators accessed with the neutral SNPs (Table 1). For the model with the outlier SNPs, only the region factor was significant to explain the average number of alleles per locus (*p* = 6.9 × 10^−6^), and the gene diversity (*p* = 4.4 × 10^−6^). Interestingly, a clear negative relationship between the percentage of forest in the landscape and the genetic estimators of *E. edulis* was observed in the BA_SR region, while no pattern was evidenced in the BA_NR region (Figure 5a,b). It is also important to note that although the percentage of forest cover interacting with the region is the most plausible model, models with richness and abundance of birds interacting with the region factor are more plausible than the null model to explain the genetic estimators accessed with the set of outlier SNPs (Table 1).

**TABLE 1:** Responses of the neutral genetic variability and outlier (putatively under selection) of *Euterpe edulis* to the abundance and richness of birds and the amount of forest cover in two regions of Atlantic Forest in Bahia, Brazil.

O‐H_E_	w_i_	0.92	0.05	0.03	0.00	0.00	0.00	0.00	0.00	0.00	0.00	0.00
	K	5	5	5	2	3	3	3	5	5	5	9
	Δ_i_	0.0	5.9	6.7	43.8	45.9	46.6	46.9	50.8	51.7	53.4	75
	Model	**H**_**E**_ ~ FC*RE	H_E_ ~ AB*RE	H_E_ ~ RI*RE	Null	H_E_ ~ FC	H_E_ ~ RI	H_E_ ~ AB	H_E_ ~ RI*AB	H_E_ ~ FC*RI	H_E_ ~ FC*AB	H_E_ ~ FC*RI*AB
N‐H_E_	w_i_	0.47	0.19	0.15	0.14	0.02	0.02	0.00	0.00	0.00	0.00	0.00
	K	2	3	3	3	5	5	5	5	5	5	**9**
	Δ_i_	0.0	1.9	2.3	2.4	6.6	7.4	8.2	8.3	9.7	10.2	37.2
	Model	**Null**	H_E_ ~ AB	H_E_ ~ RI	H_E_ ~ FC	H_E_ ~ FC*RE	H_E_ ~ RI*AB	H_E_ ~ FC*AB	H_E_ ~ FC*RI	H_E_ ~ AB*RE	H_E_ ~ RI*RE	H_E_ ~ FC*RI*AB
O‐alleles	w_i_	0.93	0.04	0.03	0.00	0.00	0.00	0.00	0.00	0.00	0.00	0.00
	K	5	5	5	2	3	3	3	5	5	5	9
	Δ_i_	0.0	6.5	6.5	44.0	45.8	46.9	47.1	51.2	52.1	53.5	75.1
	Model	A ~ FC*RE	A ~ AB*RE	A ~ RI*RE	Null	A ~ FC	A ~ RI	A ~ AB	A ~ RI*AB	A ~ FC*RI	A ~ FC*AB	A ~ FC*RI*AB
N‐alleles	w_i_	0.39	0.23	0.17	0.12	0.03	0.03	0.00	0.00	0.00	0.00	0.00
	K	2	3	3	5	5	5	5	5	5	5	9
	Δ_i_	0.0	1.0	1.7	2.3	4.8	5.4	7.7	8.0	8.0	8.6	35.3
	Model	**Null**	A ~ RI	A ~ AB	A ~ FC	A ~ FC*RE	A ~ RI*AB	A ~ RI*RE	A ~ FC*RI	A ~ FC*AB	A ~ AB*RE	A ~ FC*RI*AB

*Note*: The most plausible models are in bold and in the Figure 5.

Abbreviations: A, number of alleles; AB, bird abundance; FC, percentage of forest cover at the landscape; H_E_, gene diversity; k, number of parameter estimates in the model; N, neutral SNPs; O, outlier SNPs; RE, Study region; RI, bird richness; w_i_, Akaike weights; Δ_i_, difference in values of Akaike Information Criterion.

## 4 DISCUSSION

In the present study, we used for the first time, SNP markers inserted in transcribed regions of the *E. edulis* genome to investigate patterns of variability and genetic structure in anthropic landscapes of the Brazilian Atlantic Forest. We demonstrate that populations of *E. edulis* show patterns of structure and genetic variability that differ between geographic regions with different deforestation and biogeographic histories, suggesting that these factors are important to understand the local adaptation process of the species. We also demonstrate that the genetic variability evaluated exclusively with outlier loci responds to forest loss. The most deforested landscapes has greater genetic variability, as a possible response to selective pressures of these environments under biotic and abiotic stresses. Overall, our study not only supports previous evidence with microsatellite markers showing that anthropic landscapes can still maintain high neutral genetic variability, but also goes a step further, showing that deforestation can influence the genetic variability of functional genomic regions under selection (outlier). In this way, our findings advance our understanding of key factors influencing genetic variability in an important palm species from one of the most biodiverse and endangered biomes on the planet. Based on our findings, we recommend increasing the amount of forest and specie enrichment in more deforested Atlantic Forest landscapes (Leal et al., 2021). With this action, it would be possible to minimize the effects of deforestation on the species and increase the chances of small and isolated populations maintain genetic variability in a long term. As good sampling practices for conservation, we also suggest some reintroduction or enrichment in areas where the species has become extinct, using exclusively seeds from populations of the same target region of conservation management (Pereira et al., 2022).

### 4.1 Genetic diversity and structure

In our study, we observed high neutral genetic variability among the 22 populations of *E. edulis*, as already reported for this species using SNP markers (Brancalion et al., 2018) and microsatellites (Carvalho et al., 2017; Pereira et al., 2022; Santos et al., 2015; Soares et al., 2019). This pattern may be related to the biology and range of occurrence of *E. edulis* given that allogeneic plants pollinated and dispersed by animals and with wide geographic distribution usually present high genetic variability (Lowe et al., 2018). Conversely, the high genetic variability observed with outlier SNPs may reflect the intensity of natural selection in these populations, indicating the distinct environmental conditions to which these populations are exposed (Bragg et al., 2015; Gao et al., 2021; Rellstab et al., 2015; Schoville et al., 2012) and, certainly, adapted.

When comparing the neutral genetic variability of *E. edulis*, we observed differences only between populations inserted in SP and BA states, suggesting that the mechanisms that generate or maintain genetic variability in this species differ mainly on a macroregional scale. These populations are separated by approximately ~1300 km and this portions of Brazilian Atlantic Forest presenting a multitude of factors that differ between them, such as the climatic stability of the habitat, biogeography and anthropogenic disturbance, which can modulate the genetic variability of natural populations of *E. edulis* (Carvalho et al., 2017; Pereira et al., 2022). Thus, the current neutral genetic variability may be depends on historical factors not evaluated in the study, which modulated events of colonization and expansion of the species (Carvalho et al., 2017; Pereira et al., 2022; Schoville et al., 2012). In fact, demographic studies indicate that populations of *E. edulis* inserted in SP have higher population density and fruit production compared with populations in BA (Leal et al., 2021; Reis et al., 2000), which could contribute, even partially, to the greater genetic diversity in populations in SP.

For the outlier loci, we showed that the mean number of alleles per locus and gene diversity differ significantly between the four studied regions, evidencing a pattern of natural selection possibly modulated by environmental characteristics (Bragg et al., 2015; Gao et al., 2021; Rellstab et al., 2015; Schoville et al., 2012). As this genetic variability is located in functional regions of the genome, which theoretically can influence the survival of individuals, it is assumed that the greater genetic diversity would be needed to adapt (i.e., heterozygote advantage) the several environmental adversities faced by individuals (Scott et al., 2020; Teixeira & Huber, 2021; Wernberg et al., 2018). Thus, we believe that there is greater genetic variability in *E. edulis* populations located in environments with more environmental adversities, whether natural or anthropogenic. In particular, there are many environmental or ecological factors that exert selective pressure, driving the adaptation of organisms, such as factors related to temperature, precipitation and soil properties (Bragg et al., 2015; Gao et al., 2021; Rellstab et al., 2017). In our study, regions more deforested, such SP_SE and BA_SR region (see Figure 1), showed greater genetic variability under selection. Thus, in addition to the existing natural variation, we believe that anthropogenic disturbances also exert important selection pressures on *E. edulis* populations (Carvalho et al., 2016; Cerqueira et al., 2021; Galetti et al., 2013; Leal et al., 2021).

When comparing geographic regions within their respective states, we found greater outlier genetic variability in BA_SR, suggesting that natural selection is more intense in this location highly deforested than in BA_NR. With the reduction of forest cover, a series of changes occur in the landscape, for example, an greater penetration of light, loss of seed‐dispersing fauna and increase in seed predation by invertebrates, which negatively impact *E. edulis* populations (Carvalho et al., 2016; Cerqueira et al., 2021; Soares et al., 2015). In fact, studies carried out in the BA_SR and BA_NR region indicate that intense deforestation caused the local extinction of *E. edulis*, while microclimatic changes in deforested landscapes led to physiological changes, with a potential negative impact on the growth and survivability of young individuals (Cerqueira et al., 2021; Leal et al., 2021). For these reasons, we believe that deforestation can impose selective pressures, favoring the detection of genes under selection (i.e, outliers) and greater genetic diversity, modulating the local adaptation of *E. edulis* (Bragg et al., 2015; Feng et al., 2015). Similarly, when comparing the regions in SP, it is possible to suppose that in addition to the visible difference in the remaining forest quantity among SP_SE and SP_RA (Figure 1c), other environmental variables probably modulate the genetic variability (Brancalion et al., 2018; Carvalho et al., 2016). For example, while SP_SE is located in the interior of the state, with a marked dry season, SP_RA is located in the wet climate zone of the coast and does not have rainfall limitations throughout the year (Eisenlohr & de Oliveira‐Filho, 2015). This difference climate can directly influence the germination of *E. edulis* seeds, which are recalcitrant and highly sensitive to desiccation (Brancalion et al., 2011). Thus, populations of *E. edulis* inserted in SP_SE could suffer greater stress compared with populations in SP_RA, which could explain our findings (Ahrens et al., 2019; Bragg et al., 2015; Feng et al., 2015). However, although our results show a very evident pattern, but due to the relatively small number of populations per region, it is important to highlight that our inferences can be influenced by noise from the analyses. Furthermore, considering the complexity of the mechanisms involved in adaptive responses in natural populations, it would be expected that multiple factors would simultaneously influence our results.

The characterization of the global genetic structure for the 22 populations revealed a great genetic divergence between the states of the BA and SP, with a subsequent subdivision within of each state (Figure S1A,B). This clustering pattern may reflect several factors (e.g., biogeographic, climatic, ecological or environmental) that characterize and differentiate the gene pool in each state (Dan et al., 2020; von Takach et al., 2021). In particular, the differences among populations located in each state are more apparent from selection tests that identified 1484 outlier loci in BA and 1053 in SP, being only 187 SNPs shared between the states (Figure S2C). These results further reinforce the importance of future study with the environmental genomic association approach, combined with the functional annotation of the genes where these SNPs are located, for genomic conservation of trees in the Atlantic Forest (Sales, 2022; Santos & Gaiotto, 2020).

When estimating the genetic structure of *E. edulis* populations in the Bahia state, a clear pattern of separation among the populations inserted in distinct regions (BA_NR and BA_SR) was evidenced, regardless of the sets of markers used in the analysis (Figure 3a–d). This result can be partially driven by geographic distance, as demonstrated by the Mantel test, indicating that genetic variability is structured in space. But, taking into account that IBD does not apply to our case, it is an indication that other processes are also acting together to modulate the genetic variability of *E. edulis* (Diniz‐Filho et al., 2013). Specifically, the high level of deforestation observed mainly in BA_SR region can alter the biotic and abiotic conditions of forest remnants, and hence creating a myriad of pervasive effect on native species (Andrade et al., 2015; Benchimol, Mariano‐Neto, et al., 2017; Benchimol, Talora, et al., 2017; Morante‐Filho et al., 2016; Pessoa et al., 2017; Rocha‐Santos et al., 2016, 2017). Furthermore, this region presented greater outlier genetic variability, reinforcing the hypothesis that *E. edulis* populations are under strong selective pressure resulting from deforestation. Our findings also showed the separation of populations in BA_SR region into two groups, possibly as result of historic deforestation that can reduce gene flow and hence the sharing of the gene pool between populations (Santos et al., 2016). In contrast, the BA_NR region is more forested and composed by fragments more connected (See Figure 1a), which may facilitate gene flow between different populations of *E. edulis*, leading thus the genetic homogeneity (Santos et al., 2016). In this context, the differences observed in the gene pool among regions can be a reflect of distinct evolutionary forces resulting from pressures imposed by divergent patterns of land‐use change.

In the state of SP, we also observed a separation of regions into two groups, possibly due to the geographic distance between populations, as evidenced in our results, associated with biogeographic influences on composition of the gene pool of the species as reported in previous studies (Brancalion et al., 2018; Carvalho et al., 2016). These results indicated that these biogeographically divergent ecosystems, with their own biotic and abiotic characteristics, present divergent adaptations, promoting differentiation in the *E. edulis* gene pool (Brancalion et al., 2018). Among such characteristics, we can highlight the differences in water availability and soil composition between SP regions, which are known to influence seed germination (Brancalion et al., 2018). Indeed, these abiotic factors can exert strong selection pressure, resulting in the local adaptation of plant species populations (Bragg et al., 2015; Gao et al., 2021; Rellstab et al., 2017; von Takach et al., 2021). In this context, future genomic studies associating environmental and climatic variables, as well as the biological functions related to these loci are needed to unravel the causes of the observed differences, which is of fundamental importance to predict the ability of *E. edulis* to respond to anthropogenic environmental changes that threaten this palm species (Santos & Gaiotto, 2020).

### 4.2 Influence of forest cover and seed dispersers on genetic variability

Our findings highlighted two important patterns: (i) only genetic estimators accessed with outlier SNPs respond to deforestation and (ii) geographic location is crucial to explain the genetic variability under selection. Our results demonstrate that the negative impacts related to the current forest loss are not yet reflected in the neutral genetic variability of this species, as already evidenced in studies with microsatellite markers in the same forest fragments (Santos et al., 2015; Soares et al., 2019). Our hypothesis is that populations inserted in deforested landscapes in BA_SR can still retain high neutral genetic diversity due to seed dispersers that maintain gene flow over short distances, as already evidenced for *E. edulis* using microsatellite markers (Carvalho, Garcia, et al., 2021). Thus, we believe that maintaining gene flow over short distances could delay the loss of genetic diversity by inbreeding depression and genetic drift in small and isolated populations. However, considering that the intensification of deforestation in southern Bahia took place in the 1990s, there would have been time for a maximum of two generations, since the species has generation time of about 18 years (Carvalho, Lucas, & Côrtes, 2021; Franco & Silvertown, 2004; Galetti et al., 2013). Therefore, we believe that populations inserted in landscapes with a low percentage of forest may suffer a loss of genetic diversity in the short – medium term, if these populations remain small and isolated (Borges et al., 2020; Lowe et al., 2015; Santos et al., 2015; Soares et al., 2019). On the other hand, we did not find evidence that landscape‐scale forest cover has influenced patterns of neutral or outlier genetic variability in the BA_NR region. It is important to highlight in this context that the BA_NR region has large fragments that are highly connected, indicating that there was no large targeted deforestation associated with the great similarity between populations, as evidenced by its genetic structure (Figure 3a–d). Therefore, we believe that this region behaves as a metapopulation, supporting the gene flow and generating genetic homogenization independent of forest cover in the landscape (Santos et al., 2016).

Interestingly, the results obtained for the outlier loci showed a negative effect of forest cover on number of alleles and gene diversity only in the BA_SR region, indicating a possible process of natural selection driven by deforestation. Thus, we believe that *E. edulis* populations inserted in more deforested landscapes have to deal with more and greater environmental adversities, which could require different adaptations, potentially explaining the greater number of alleles and diversity under selection (Estravis‐Barcala et al., 2020; Markert et al., 2010; Reed & Frankham, 2003; Scott et al., 2020; Wernberg et al., 2018). Another aspect that deserves attention is the fact that forest loss influences only the SNPs identified as outliers, as demonstrated by our selection of models (Table 1). A plausible hypothesis for this pattern would be a rapid adaptive response of the population to the physiological stress that is known to occur in deforested landscapes in BA_SR (Cerqueira et al., 2021). Thus, considering that these SNPs are inserted in transcribed regions of the genome that theoretically can influence the survival of individuals, it would be expected that alleles of genes associated with responses to physiological stresses would increase in frequency in the population (i.e, outlier SNPs). On the other hand, if neutral SNPs are located in gene regions that are not associated with responses to the stresses that populations are currently exposed to, changes in genetic variability due to deforestation could be delayed by gene flow over short distances. This difference in the response of neutral and outlier SNPs to deforestation suggests that caution is needed in conservationist inferences when no influence of anthropic actions is detected under the neutral genetic variability of small and isolated populations (Teixeira & Huber, 2021). However, it is important to note that these results are based on 4252 neutral SNPs and 1484 outliers, evaluating only seven populations in BA_SR and nine in BA_NR, which could generate noise in our results, in addition to the need to experimentally validate this hypothesis.

In general, the landscape genomics approach used in the present study has advanced the understanding of genetic responses of perennial species dispersed and pollinated by animals in a scenario of rapid anthropogenic environmental changes. Thus, our results can assist in decision‐making aimed at efficient strategies for the conservation of a target species as well as in predicting the responses of other perennial species with similar biology facing anthropogenic disturbances. In addition, our findings reveal the genomic effects caused by forest loss in tropical environments. They make it possible to predict the consequences of disturbances on genomic variability and, consequently, on the adaptive potential of species.

## CONFLICT OF INTEREST

The authors have no conflicts of interest to declare.

## Supporting information


Appendix S1.

## Data Availability

The files containing the sampling locations, the 7632 SNP markers identified and used in the study, the subset containing the neutral SNP markers and the subset with the SNP markers identified as outliers will be deposited in the Dryad database.

## References

[eva13525-bib-0001] Ahrens, C. W. , Rymer, P. D. , Stow, A. , Bragg, J. , Dillon, S. , Umbers, K. D. , & Dudaniec, R. Y. (2018). The search for loci under selection: Trends, biases and progress. Molecular Ecology, 27(6), 1342–1356. 29524276 10.1111/mec.14549

[eva13525-bib-0002] Anderson, D. R. (2008). Model based inference in the life sciences: A primer on evidence. Springer.

[eva13525-bib-0003] Andrade, E. R. , Jardim, J. G. , Santos, B. A. , Melo, F. P. L. , Talora, D. C. , Faria, D. , & Cazetta, E. (2015). Effects of habitat loss on taxonomic and phylogenetic diversity of understory Rubiaceae in Atlantic forest landscapes. Forest Ecology and Management, 349, 73–84.

[eva13525-bib-0004] Arroyo‐Rodríguez, V. , Fahrig, L. , Tabarelli, M. , Watling, J. I. , Tischendorf, L. , Benchimol, M. , Cazetta, E. , Faria, D. , Leal, I. R. , Melo, F. P. L. , Morante‐Filho, J. C. , Santos, B. A. , Arasa‐Gisbert, R. , Arce‐Peña, N. , Cervantes‐López, M. J. , Cudney‐Valenzuela, S. , Galán‐Acedo, C. , San‐José, M. , Vieira, I. C. G. , … Tscharntke, T. (2020). Designing optimal human‐modified landscapes for forest biodiversity conservation. Ecology Letters, 23(9), 1404–1420. 32537896 10.1111/ele.13535

[eva13525-bib-0005] Benchimol, M. , Mariano‐Neto, E. , Faria, D. , Rocha‐Santos, L. , de Souza Pessoa, M. , Gomes, F. S. , Talora, D. C. , & Cazetta, E. (2017). Translating plant community responses to habitat loss into conservation practices: Forest cover matters. Biological Conservation, 209, 499–507.

[eva13525-bib-0006] Benchimol, M. , Talora, D. C. , Mariano‐Neto, E. , Oliveira, T. L. , Leal, A. , Mielke, M. S. , & Faria, D. (2017). Losing our palms: The influence of landscape‐scale deforestation on Arecaceae diversity in the Atlantic forest. Forest Ecology and Management, 384, 314–322.

[eva13525-bib-0007] Benestan, L. M. , Ferchaud, A. L. , Hohenlohe, P. A. , Garner, B. A. , Naylor, G. J. , Baums, I. B. , Schwartz, M. K. , Kelley, J. L. , & Luikart, G. (2016). Conservation genomics of natural and managed populations: Building a conceptual and practical framework. Molecular Ecology, 25(13), 2967–2977. 27086132 10.1111/mec.13647

[eva13525-bib-0008] Bolker, B. M. (2012). Bbmle: Tools for general maximum likelihood estimation. R package version 1.0.15. R Foundation for Statistical Computing. https://cran.r‐project.org/web/packages/bbmle/index.html

[eva13525-bib-0009] Borges, D. B. , Mariano‐Neto, E. , Caribé, D. S. , Corrêa, R. X. , & Gaiotto, F. A. (2020). Changes in fine‐scale spatial genetic structure related to protection status in Atlantic rain Forest fragment. Journal for Nature Conservation, 53, 125784.

[eva13525-bib-0010] Bragg, J. G. , Supple, M. A. , Andrew, R. L. , & Borevitz, J. O. (2015). Genomic variation across landscapes: Insights and applications. New Phytologist, 207(4), 953–967. 25904408 10.1111/nph.13410

[eva13525-bib-0011] Brancalion, P. H. , Oliveira, G. C. , Zucchi, M. I. , Novello, M. , van Melis, J. , Zocchi, S. S. , Chazdon, R. L. , & Rodrigues, R. R. (2018). Phenotypic plasticity and local adaptation favor range expansion of a neotropical palm. Ecology and Evolution, 8(15), 7462–7475. 30151163 10.1002/ece3.4248 PMC6106193

[eva13525-bib-0118] Brancalion, P. H. S. A. , Novembre, A. D. D. L. C. , & Rodrigues, R. R. (2011). Seed development, yield and quality of two palm species growing in different tropical forest types in SE Brazil: implications for ecological restoration. Seed Science and Technology, 39(2), 412–424.

[eva13525-bib-0012] Brancalion, P. H. , Vidal, E. , Lavorenti, N. A. , Batista, J. L. F. , & Rodrigues, R. R. (2012). Soil‐mediated effects on potential *Euterpe edulis* (Arecaceae) fruit and palm heart sustainable management in the Brazilian Atlantic Forest. Forest Ecology and Management, 284, 78–85.

[eva13525-bib-0013] Ćalić, I. , Bussotti, F. , Martínez‐García, P. J. , & Neale, D. B. (2016). Recent landscape genomics studies in forest trees—What can we believe? Tree Genetics & Genomes, 12(1), 3.

[eva13525-bib-0014] Carvalho, C. D. S. , Garcia, C. , Lucas, M. S. , Jordano, P. , & Cortes, M. C. (2021). Extant fruit‐eating birds promote genetically diverse seed rain, but disperse to fewer sites in defaunated tropical forests. Journal of Ecology, 109(2), 1055–1067.

[eva13525-bib-0015] Carvalho, C. D. S. , Lucas, M. S. , & Côrtes, M. C. (2021). Rescuing intraspecific variation in human‐impacted environments. Journal of Applied Ecology, 58(2), 350–359.

[eva13525-bib-0016] Carvalho, C. S. , Ballesteros‐Mejia, L. , Ribeiro, M. C. , Côrtes, M. C. , Santos, A. S. , & Collevatti, R. G. (2017). Climatic stability and contemporary human impacts affect the genetic diversity and conservation status of a tropical palm in the Atlantic Forest of Brazil. Conservation Genetics, 18(2), 467–478.

[eva13525-bib-0017] Carvalho, C. S. , Galetti, M. , Colevatti, R. G. , & Jordano, P. (2016). Defaunation leads to microevolutionary changes in a tropical palm. Scientific Reports, 6(1), 31957. 27535709 10.1038/srep31957 PMC4989191

[eva13525-bib-0018] Carvalho, C. S. , Ribeiro, M. C. , Côrtes, M. C. , Galetti, M. , & Collevatti, R. G. (2015). Contemporary and historic factors influence differently genetic differentiation and diversity in a tropical palm. Heredity, 115(3), 216–224. 25873150 10.1038/hdy.2015.30 PMC4814231

[eva13525-bib-0019] Cerqueira, A. F. , Rocha‐Santos, L. , Benchimol, M. , & Mielke, M. S. (2021). Habitat loss and canopy openness mediate leaf trait plasticity of an endangered palm in the Brazilian Atlantic Forest. Oecologia, 196(3), 619–631. 33630171 10.1007/s00442-021-04879-x

[eva13525-bib-0020] Chen, H. , & Boutros, P. C. (2011). VennDiagram: A package for the generation of highly‐customizable Venn and Euler diagrams in R. BMC Bioinformatics, 12(1), 1–7. 21269502 10.1186/1471-2105-12-35 PMC3041657

[eva13525-bib-0021] Cockerham, C. C. , & Weir, B. S. (1987). Correlations, descent measures: Drift with migration and mutation. Proceedings of the National Academy of Sciences, 84(23), 8512–8514. 10.1073/pnas.84.23.8512 PMC299574 3479805

[eva13525-bib-0022] Curtis, P. G. , Slay, C. M. , Harris, N. L. , Tyukavina, A. , & Hansen, M. C. (2018). Classifying drivers of global forest loss. Science, 361(6407), 1108–1111. 30213911 10.1126/science.aau3445

[eva13525-bib-0023] Dan, C. X. , Yang, J. , Guo, Y. F. , Zhao, Y. M. , Zhou, T. , Zhang, X. , Ju, M. M. , Li, Z. H. , & Zhao, G. (2020). Spatial genetic structure and demographic history of the dominant forest oak *Quercus fabri* Hance in subtropical China. Frontiers in Plant Science, 11, 2278. 10.3389/fpls.2020.583284 PMC7889815 33613578

[eva13525-bib-0024] Danecek, P. , Auton, A. , Abecasis, G. , Albers, C. A. , Banks, E. , DePristo, M. A. , Handsaker, R. E. , Lunter, G. , Marth, G. T. , Sherry, S. T. , McVean, G. , Durbin, R. , & 1000 Genomes Project Analysis Group . (2011). The variant call format and VCFtools. Bioinformatics, 27(15), 2156–2158. 21653522 10.1093/bioinformatics/btr330 PMC3137218

[eva13525-bib-0025] De Andrade, A. C. S. (2001). The effect of moisture content and temperature on the longevity of heart of palm seeds (*Euterpe edulis*). Seed Science and Technology, 29(1), 171–182.

[eva13525-bib-0026] Dean, W. (1996). A ferro e fogo: a história e a devastação da Mata Atlântica brasileira (Vol. 2004, p. 484). Cia. das Letras.

[eva13525-bib-0027] Diniz‐Filho, J. A. F. , Soares, T. N. , Lima, J. S. , Dobrovolski, R. , Landeiro, V. L. , de Campos Telles, M. P. , Rangel, T. F. , & Bini, L. M. (2013). Mantel test in population genetics. Genetics and Molecular Biology, 36, 475–485. 24385847 10.1590/S1415-47572013000400002 PMC3873175

[eva13525-bib-0028] Doyle, J. J. , & Doyle, J. L. (1990). Isolation of plant DNA from fresh tissue. Focus, 12(13), 39–40.

[eva13525-bib-0029] Eisenlohr, P. V. , & de Oliveira‐Filho, A. T. (2015). Revisiting patterns of tree species composition and their driving forces in the Atlantic forests of southeastern Brazil. Biotropica, 47(6), 689–701.

[eva13525-bib-0030] Estravis‐Barcala, M. , Mattera, M. G. , Soliani, C. , Bellora, N. , Opgenoorth, L. , Heer, K. , & Arana, M. V. (2020). Molecular bases of responses to abiotic stress in trees. Journal of Experimental Botany, 71(13), 3765–3779. 31768543 10.1093/jxb/erz532 PMC7316969

[eva13525-bib-0032] Fariello, M. I. , Boitard, S. , Naya, H. , SanCristobal, M. , & Servin, B. (2013). Detecting signatures of selection through haplotype differentiation among hierarchically structured populations. Genetics, 193(3), 929–941. 23307896 10.1534/genetics.112.147231 PMC3584007

[eva13525-bib-0033] Feng, X. J. , Jiang, G. F. , & Fan, Z. (2015). Identification of outliers in a genomic scan for selection along environmental gradients in the bamboo locust, *Ceracris kiangsu* . Scientific Reports, 5(1), 1–11. 10.1038/srep13758 PMC4558720 26333424

[eva13525-bib-0034] Fischer, M. C. , Rellstab, C. , Tedder, A. , Zoller, S. , Gugerli, F. , Shimizu, K. K. , Holderegger, R. , & Widmer, A. (2013). Population genomic footprints of selection and associations with climate in natural populations of *Arabidopsis halleri* from the Alps. Molecular Ecology, 22(22), 5594–5607. 24102711 10.1111/mec.12521 PMC4274019

[eva13525-bib-0035] Fitzpatrick, M. C. , & Keller, S. R. (2015). Ecological genomics meets community‐level modelling of biodiversity: Mapping the genomic landscape of current and future environmental adaptation. Ecology Letters, 18(1), 1–16. 25270536 10.1111/ele.12376

[eva13525-bib-0036] Forester, B. R. , Jones, M. R. , Joost, S. , Landguth, E. L. , & Lasky, J. R. (2016). Detecting spatial genetic signatures of local adaptation in heterogeneous landscapes. Molecular Ecology, 25(1), 104–120. 26576498 10.1111/mec.13476

[eva13525-bib-0037] Franco, M. , & Silvertown, J. (2004). A comparative demography of plants based upon elasticities of vital rates. Ecology, 85, 531–538.

[eva13525-bib-0038] Frichot, E. , & François, O. (2015). LEA: An R package for landscape and ecological association studies. Methods in Ecology and Evolution, 6(8), 925–929.

[eva13525-bib-0039] Frichot, E. , Schoville, S. D. , Bouchard, G. , & François, O. (2013). Testing for associations between loci and environmental gradients using latent factor mixed models. Molecular Biology and Evolution, 30(7), 1687–1699. 23543094 10.1093/molbev/mst063 PMC3684853

[eva13525-bib-0040] Gaiotto, F. A. , Grattapaglia, D. , & Vencovsky, R. (2003). Genetic structure, mating system, and long‐distance gene *fl*ow in heart of palm (*Euterpe edulis* Mart.). The Journal of Heredity, 94, 399–406. 14557393 10.1093/jhered/esg087

[eva13525-bib-0041] Galetti, M. , Guevara, R. , Côrtes, M. C. , Fadini, R. , Von Matter, S. , Leite, A. B. , Labecca, F. , Ribeiro, T. , Carvalho, C. S. , Collevatti, R. G. , Pires, M. M. , Guimarães, P. R., Jr. , Brancalion, P. H. , Ribeiro, M. C. , & Jordano, P. (2013). Functional extinction of birds drives rapid evolutionary changes in seed size. Science, 340(6136), 1086–1090. 23723235 10.1126/science.1233774

[eva13525-bib-0043] Gao, J. , Liu, Z. L. , Zhao, W. , Tomlinson, K. W. , Xia, S. W. , Zeng, Q. Y. , Wang, X.‐R. , & Chen, J. (2021). Combined genotype and phenotype analyses reveal patterns of genomic adaptation to local environments in the subtropical oak *Quercus acutissima* . Journal of Systematics and Evolution, 59(3), 541–556.

[eva13525-bib-0044] Garrison, E. , & Marth, G. (2012). Haplotype‐based variant detection from short‐read sequencing. arXiv 2012. *arXiv preprint arXiv:1207.3907*.

[eva13525-bib-0046] Gautier, M. (2015). Genome‐wide scan for adaptive differentiation and association analysis with population‐specific covariables. Genetics, 201(4), 1555–1579. 26482796 10.1534/genetics.115.181453 PMC4676524

[eva13525-bib-0048] González, A. V. , Gómez‐Silva, V. , Ramírez, M. J. , & Fontúrbel, F. E. (2020). Meta‐analysis of the differential effects of habitat fragmentation and degradation on plant genetic diversity. Conservation Biology, 34(3), 711–720. 31605401 10.1111/cobi.13422

[eva13525-bib-0049] Goslee, S. C. , & Urban, D. L. (2007). The ecodist package for dissimilarity‐based analysis of ecological data. Journal of Statistical Software, 22, 1–19.

[eva13525-bib-0050] Günther, T. , & Coop, G. (2013). Robust identification of local adaptation from allele frequencies. Genetics, 195(1), 205–220. 23821598 10.1534/genetics.113.152462 PMC3761302

[eva13525-bib-0052] Hivert, V. , Leblois, R. , Petit, E. J. , Gautier, M. , & Vitalis, R. (2018). Measuring genetic differentiation from pool‐seq data. Genetics, 210(1), 315–330. 30061425 10.1534/genetics.118.300900 PMC6116966

[eva13525-bib-0053] Holbrook, K. M. (2011). Home range and movement patterns of toucans: Implications for seed dispersal. Biotropica, 43(3), 357–364.

[eva13525-bib-0054] Holderegger, R. , Buehler, D. , Gugerli, F. , & Manel, S. (2010). Landscape genetics of plants. Trends in Plant Science, 15(12), 675–683. 20940103 10.1016/j.tplants.2010.09.002

[eva13525-bib-0055] Jombart, T. , & Ahmed, I. (2011). Adegenet 1.3‐1: New tools for the analysis of genome‐wide SNP data. Bioinformatics, 27(21), 3070–3071. 21926124 10.1093/bioinformatics/btr521 PMC3198581

[eva13525-bib-0057] Lanes, É. C. , Pope, N. S. , Alves, R. , Carvalho Filho, N. M. , Giannini, T. C. , Giulietti, A. M. , Imperatriz‐Fonseca, V. L. , Monteiro, W. , Oliveira, G. , Silva, A. R. , Siqueira, J. O. , Souza‐Filho, P. W. , Vasconcelos, S. , & Jaffé, R. (2018). Landscape genomic conservation assessment of a narrow‐endemic and a widespread morning glory from Amazonian savannas. Frontiers in Plant Science, 9, 532. 29868042 10.3389/fpls.2018.00532 PMC5949356

[eva13525-bib-0058] Leal, A. , Benchimol, M. , Faria, D. , Dodonov, P. , & Cazetta, E. (2021). Landscape‐scale forest loss shapes demographic structure of the threatened tropical palm *Euterpe edulis* mart.(Arecaceae). Forest Ecology and Management, 502, 119716.

[eva13525-bib-0059] Lee, W. P. , Stromberg, M. P. , Ward, A. , Stewart, C. , Garrison, E. P. , & Marth, G. T. (2014). MOSAIK: A hash‐based algorithm for accurate next‐generation sequencing short‐read mapping. PLoS One, 9(3), e90581. 24599324 10.1371/journal.pone.0090581 PMC3944147

[eva13525-bib-0060] Lima, M. M. , & Mariano‐Neto, E. (2014). Extinction thresholds for Sapotaceae due to forest cover in Atlantic Forest landscapes. Forest Ecology and Management, 312, 260–270.

[eva13525-bib-0061] Lotterhos, K. E. , & Whitlock, M. C. (2015). The relative power of genome scans to detect local adaptation depends on sampling design and statistical method. Molecular Ecology, 24(5), 1031–1046. 25648189 10.1111/mec.13100

[eva13525-bib-0062] Lowe, A. J. , Breed, M. F. , Caron, H. , Colpaert, N. , Dick, C. , Finegan, B. , Gardner, M. , Gheysen, G. , Gribel, R. , Harris, J. B. C. , Kremer, A. , Lemes, M. R. , Margis, R. , Navarro, C. M. , Salgueiro, F. , Villalobos‐Barrantes, H. M. , & Cavers, S. (2018). Standardized genetic diversity‐life history correlates for improved genetic resource management of neotropical trees. Diversity and Distributions, 24(6), 730–741.

[eva13525-bib-0063] Lowe, A. J. , Cavers, S. , Boshier, D. , Breed, M. F. , & Hollingsworth, P. M. (2015). The resilience of forest fragmentation genetics—no longer a paradox—We were just looking in the wrong place. Heredity, 115(2), 97–99. 26176685 10.1038/hdy.2015.40 PMC4815445

[eva13525-bib-0064] Luu, K. , Bazin, E. , & Blum, M. G. (2017). Pcadapt: An R package to perform genome scans for selection based on principal component analysis. Molecular Ecology Resources, 17(1), 67–77. 27601374 10.1111/1755-0998.12592

[eva13525-bib-0066] Manel, S. , Andrello, M. , Henry, K. , Verdelet, D. , Darracq, A. , Guerin, P. E. , Desprez, B. , & Devaux, P. (2018). Predicting genotype environmental range from genome–environment associations. Molecular Ecology, 27(13), 2823–2833. 29772088 10.1111/mec.14723

[eva13525-bib-0067] Manel, S. , Joost, S. , Epperson, B. K. , Holderegger, R. , Storfer, A. , Rosenberg, M. S. , Scribner, K. T. , Bonin, A. , & Fortin, M. J. (2010). Perspectives on the use of landscape genetics to detect genetic adaptive variation in the field. Molecular Ecology, 19(17), 3760–3772. 20723056 10.1111/j.1365-294X.2010.04717.x

[eva13525-bib-0068] Mantel, N. (1967). The detection of disease clustering and a generalized regression approach. Cancer Research, 27(2 Part 1), 209–220. 6018555

[eva13525-bib-0069] Markert, J. A. , Champlin, D. M. , Gutjahr‐Gobell, R. , Grear, J. S. , Kuhn, A. , McGreevy, T. J. , Roth, A. , Bagley, M. J. , & Nacci, D. E. (2010). Population genetic diversity and fitness in multiple environments. BMC Evolutionary Biology, 10(1), 1–13. 20609254 10.1186/1471-2148-10-205 PMC2927917

[eva13525-bib-0070] Martinelli, M. , & Moraes, A. (2013). Livro vermelho da flora do Brasil (p. 1100). Instituto de Pesquisas Jardim Botânico do Rio de Janeiro.

[eva13525-bib-0071] Martini, A. M. Z. , Fiaschi, P. , Amorim, A. M. , & Da Paixão, J. L. (2007). A hot‐point within a hot‐spot: A high diversity site in Brazil's Atlantic Forest. Biodiversity and Conservation, 16(11), 3111–3128.

[eva13525-bib-0072] Moran, C. , & Catterall, C. P. (2014). Responses of seed‐dispersing birds to amount of rainforest in the landscape around fragments. Conservation Biology, 28(2), 551–560. 24548306 10.1111/cobi.12236

[eva13525-bib-0074] Morante‐Filho, J. C. , Arroyo‐Rodríguez, V. , & Faria, D. (2016). Patterns and predictors of β‐diversity in the fragmented Brazilian Atlantic forest: A multiscale analysis of forest specialist and generalist birds. Journal of Animal Ecology, 85(1), 240–250. 26399774 10.1111/1365-2656.12448

[eva13525-bib-0075] Morante‐Filho, J. C. , Faria, D. , Mariano‐Neto, E. , & Rhodes, J. (2015). Birds in anthropogenic landscapes: The responses of ecological groups to forest loss in the Brazilian Atlantic Forest. PLoS One, 10(6), e0128923. 26083245 10.1371/journal.pone.0128923 PMC4471271

[eva13525-bib-0076] Muler, A. E. , Rother, D. C. , Brancalion, P. S. , Naves, R. P. , Rodrigues, R. R. , & Pizo, M. A. (2014). Can overharvesting of a non‐timber‐forest‐product change the regeneration dynamics of a tropical rainforest? The case study of *Euterpe edulis* . Forest Ecology and Management, 324, 117–125.

[eva13525-bib-0077] Myers, N. , Mittermeier, R. A. , Mittermeier, C. G. , Da Fonseca, G. A. , & Kent, J. (2000). Biodiversity hotspots for conservation priorities. Nature, 403(6772), 853–858. 10706275 10.1038/35002501

[eva13525-bib-0078] Nei, M. (1978). Estimation of average heterozygosity and genetic distance from a small number of individuals. Genetics, 89(3), 583–590. 17248844 10.1093/genetics/89.3.583 PMC1213855

[eva13525-bib-0079] Orr, H. A. (2005). The genetic theory of adaptation: A brief history. Nature Reviews Genetics, 6(2), 119–127. 10.1038/nrg1523 15716908

[eva13525-bib-0080] Parisod, C. , & Holderegger, R. (2012). Adaptive landscape genetics: Pitfalls and benefits. Molecular Ecology, 21(15), 3644–3646. 22988574 10.1111/j.1365-294x.2012.05675.x

[eva13525-bib-0081] Pereira, A. G. , da Silva Ferreira, M. F. , da Silveira, T. C. , Soler‐Guilhen, J. H. , Canal, G. B. , Alves, L. B. , de Almeida, F. A. N. , Gaiotto, F. A. , & Ferreira, A. (2022). Patterns of genetic diversity and structure of a threatened palm species (*Euterpe edulis* Arecaceae) from the Brazilian Atlantic Forest. Heredity, 129(3), 161–168. 35697755 10.1038/s41437-022-00549-7 PMC9411632

[eva13525-bib-0082] Pessoa, M. S. , Hambuckers, A. , Benchimol, M. , Rocha‐Santos, L. , Bomfim, J. A. , Faria, D. , & Cazetta, E. (2017). Deforestation drives functional diversity and fruit quality changes in a tropical tree assemblage. Perspectives in Plant Ecology, Evolution and Systematics, 28, 78–86.

[eva13525-bib-0083] QGIS Development Team . (2021). QGIS geographic information system. Open Source Geospatial Foundation Project. https://www.qgis.org

[eva13525-bib-0084] R Core Team . (2021). R: A language and environment for statistical computing. R Foundation for Statistical Computing. https://www.R‐project.org

[eva13525-bib-0085] Reed, D. H. , & Frankham, R. (2003). Correlation between fitness and genetic diversity. Conservation Biology, 17(1), 230–237.

[eva13525-bib-0086] Reis, M. S. , Fantini, A. C. , Nodari, R. O. , Reis, A. , Guerra, M. P. , & Mantovani, A. (2000). Management and conservation of natural populations in Atlantic rain Forest: The case study of palm heart (*Euterpe edulis* Martius). Biotropica, 32, 894–902.

[eva13525-bib-0087] Rellstab, C. , Fischer, M. C. , Zoller, S. , Graf, R. , Tedder, A. , Shimizu, K. K. , Widmer, A. , Holderegger, R. , & Gugerli, F. (2017). Local adaptation (mostly) remains local: Reassessing environmental associations of climate‐related candidate SNPs in *Arabidopsis halleri* . Heredity, 118(2), 193–201. 27703154 10.1038/hdy.2016.82 PMC5234484

[eva13525-bib-0088] Rellstab, C. , Gugerli, F. , Eckert, A. J. , Hancock, A. M. , & Holderegger, R. (2015). A practical guide to environmental association analysis in landscape genomics. Molecular Ecology, 24(17), 4348–4370. 26184487 10.1111/mec.13322

[eva13525-bib-0089] Rezende, C. L. , Scarano, F. R. , Assad, E. D. , Joly, C. A. , Metzger, J. P. , Strassburg, B. B. N. , Tabarelli, M. , Fonseca, G. A. , & Mittermeier, R. A. (2018). From hotspot to hopespot: An opportunity for the Brazilian Atlantic Forest. Perspectives in Ecology and Conservation, 16(4), 208–214.

[eva13525-bib-0090] Rocha, L. B. (2006). *A região cacaueira da Bahia: uma abordagem fenomenológica* [Thesis]. Universidade Federal de Sergipe.

[eva13525-bib-0092] Rocha‐Santos, L. , Benchimol, M. , Mayfield, M. M. , Faria, D. , Pessoa, M. S. , Talora, D. C. , Mariano‐Neto, E. , & Cazetta, E. (2017). Functional decay in tree community within tropical fragmented landscapes: Effects of landscape‐scale forest cover. PLoS One, 12(4), e0175545. 28403166 10.1371/journal.pone.0175545 PMC5389823

[eva13525-bib-0093] Rocha‐Santos, L. , Pessoa, M. S. , Cassano, C. R. , Talora, D. C. , Orihuela, R. L. , Mariano‐Neto, E. , Morante Filho, J. C. , Faria, D. , & Cazetta, E. (2016). The shrinkage of a forest: Landscape‐scale deforestation leading to overall changes in local forest structure. Biological Conservation, 196, 1–9.

[eva13525-bib-0094] Rousset, F. (1997). Genetic differentiation and estimation of gene flow from F‐statistics under isolation by distance. Genetics, 145(4), 1219–1228. 9093870 10.1093/genetics/145.4.1219 PMC1207888

[eva13525-bib-0119] Sales, C. M. M. (2022). Descobrindo as funções para genes sob seleção em florestas tropicais antropizadas: um estudo de caso em Euterpe edulis (Arecaceae) [Thesis]. Universidade Estadual de Santa Cruz.

[eva13525-bib-0095] Santos, A. S. , Cazetta, E. , Dodonov, P. , Faria, D. , & Gaiotto, F. A. (2016). Landscape‐scale deforestation decreases gene flow distance of a keystone tropical palm, *Euterpe edulis* Mart (Arecaceae). Ecology and Evolution, 6(18), 6586–6598. 27777732 10.1002/ece3.2341 PMC5058530

[eva13525-bib-0096] Santos, A. S. , Cazetta, E. , Morante Filho, J. C. , Baumgarten, J. , Faria, D. , & Gaiotto, F. A. (2015). Lessons from a palm: Genetic diversity and structure in anthropogenic landscapes from Atlantic Forest, Brazil. Conservation Genetics, 16(6), 1295–1302.

[eva13525-bib-0097] Santos, A. S. , & Gaiotto, F. A. (2020). Knowledge status and sampling strategies to maximize cost‐benefit ratio of studies in landscape genomics of wild plants. Scientific Reports, 10(1), 1–9. 32111897 10.1038/s41598-020-60788-8 PMC7048820

[eva13525-bib-0098] Santos, J. , Varassin, I. G. , Muschner, V. C. , & Ovaskainen, O. (2018). Estimating seed and pollen dispersal kernels from genetic data demonstrates a high pollen dispersal capacity for an endangered palm species. American Journal of Botany, 105, 1802–1812. 30347429 10.1002/ajb2.1176

[eva13525-bib-0099] Schlötterer, C. , Tobler, R. , Kofler, R. , & Nolte, V. (2014). Sequencing pools of individuals—Mining genome‐wide polymorphism data without big funding. Nature Reviews Genetics, 15(11), 749–763. 10.1038/nrg3803 25246196

[eva13525-bib-0100] Schoville, S. D. , Bonin, A. , François, O. , Lobreaux, S. , Melodelima, C. , & Manel, S. (2012). Adaptive genetic variation on the landscape: Methods and cases. Annual Review of Ecology, Evolution, and Systematics, 43, 23–43.

[eva13525-bib-0101] Scott, P. A. , Allison, L. J. , Field, K. J. , Averill‐Murray, R. C. , & Shaffer, H. B. (2020). Individual heterozygosity predicts translocation success in threatened desert tortoises. Science, 370(6520), 1086–1089. 33243888 10.1126/science.abb0421

[eva13525-bib-0102] Silva, M. D. G. C. , Martini, A. M. , & Araújo, Q. R. D. (2009). Estrutura populacional de *Euterpe edulis* Mart. no sul da Bahia, Brasil. Brazilian Journal of Botany, 32(2), 393–403.

[eva13525-bib-0103] Soares, L. A. S. S. , Cazetta, E. , Santos, L. R. , França, D. D. S. , & Gaiotto, F. A. (2019). Anthropogenic disturbances eroding the genetic diversity of a threatened palm tree: A multiscale approach. Frontiers in Genetics, 10, 1090. 31788000 10.3389/fgene.2019.01090 PMC6855268

[eva13525-bib-0104] Soares, L. A. S. S. , Faria, D. , Vélez‐Garcia, F. , Vieira, E. M. , Talora, D. C. , & Cazetta, E. (2015). Implications of habitat loss on seed predation and early recruitment of a keystone palm in anthropogenic landscapes in the Brazilian Atlantic rainforest. PLoS One, 10(7), e0133540. 26186339 10.1371/journal.pone.0133540 PMC4505908

[eva13525-bib-0105] Sork, V. L. (2018). Genomic studies of local adaptation in natural plant populations. Journal of Heredity, 109(1), 3–15. 10.1093/jhered/esx091 29045754

[eva13525-bib-0106] Steane, D. A. , Potts, B. M. , McLean, E. , Prober, S. M. , Stock, W. D. , Vaillancourt, R. E. , & Byrne, M. (2014). Genome‐wide scans detect adaptation to aridity in a widespread forest tree species. Molecular Ecology, 23(10), 2500–2513. 24750317 10.1111/mec.12751

[eva13525-bib-0107] Storfer, A. , Murphy, M. A. , Spear, S. F. , Holderegger, R. , & Waits, L. P. (2010). Landscape genetics: Where are we now? Molecular Ecology, 19(17), 3496–3514. 20723061 10.1111/j.1365-294X.2010.04691.x

[eva13525-bib-0108] Teixeira, J. C. , & Huber, C. D. (2021). The inflated significance of neutral genetic diversity in conservation genetics. Proceedings of the National Academy of Sciences of the United States of America, 118(10), e2015096118. 33608481 10.1073/pnas.2015096118 PMC7958437

[eva13525-bib-0109] Thomas, W. W. , de Andre Carvalho, M. V. , Andre Amorim, M. A. , Garrison, J. , & Arbela, A. L. (1998). Plant endemism in two forests in southern Bahia, Brazil. Biodiversity and Conservation, 7(3), 311–322.

[eva13525-bib-0110] von Takach, B. , Ahrens, C. W. , Lindenmayer, D. B. , & Banks, S. C. (2021). Scale‐dependent signatures of local adaptation in a foundation tree species. Molecular Ecology, 30(10), 2248–2261. 33740830 10.1111/mec.15894

[eva13525-bib-0111] Vranckx, G. U. Y. , Jacquemyn, H. , Muys, B. , & Honnay, O. (2012). Meta‐analysis of susceptibility of woody plants to loss of genetic diversity through habitat fragmentation. Conservation Biology, 26(2), 228–237. 22044646 10.1111/j.1523-1739.2011.01778.x

[eva13525-bib-0112] Wernberg, T. , Coleman, M. A. , Bennett, S. , Thomsen, M. S. , Tuya, F. , & Kelaher, B. P. (2018). Genetic diversity and kelp forest vulnerability to climatic stress. Scientific Reports, 8(1), 1–8. 29382916 10.1038/s41598-018-20009-9 PMC5790012

[eva13525-bib-0113] Wickham, H. (2016). ggplot2: Elegant graphics for data analysis. Springer.

[eva13525-bib-0114] Wright, S. (1931). Evolution in mendelian populations. Genetics, 16(2), 97–159. 17246615 10.1093/genetics/16.2.97 PMC1201091

[eva13525-bib-0116] Zhao, S. , Guo, Y. , Sheng, Q. , & Shyr, Y. (2014). Heatmap3: An improved heatmap package with more powerful and convenient features. BMC Bioinformatics, 15(10), 1–2.24383880

[eva13525-bib-0117] Zurbuchen, A. , Landert, L. , Klaiber, J. , Müller, A. , Hein, S. , & Dorn, S. (2010). Maximum foraging ranges in solitary bees: Only few individuals have the capability to cover long foraging distances. Biological Conservation, 143(3), 669–676.

