# Supplementary material for: Tropical forest loss and geographic location drive the functional genomic diversity of an endangered palm tree
Source: Evol Appl. 2023 Jun 15;16(7):1257–73. doi: 10.1111/eva.13525 (PMC10363835; doi:10.1111/eva.13525)
Supplement: Supplementary file 1 — Appendix S1. [file EVA-16-1257-s001.docx]

**Supplemental Information**

**Tropical forest loss and geographic location drive the functional genomic diversity of an endangered palm tree**

**Table S1.**  Description of the forest cover, biological variables, SNPs markers used and estimates of genetic variability for 22 populations of *E. edulis* sampled in Atlantic Forest fragments located in the states of Bahia (BA) and São Paulo (SP), Brazil.

| Population | Nº | FC | RI | AB | N-SNPs | O-SNPs | N-Alleles | O-Alleles | N-H_E_ | O-H_E_ |
| --- | --- | --- | --- | --- | --- | --- | --- | --- | --- | --- |
| BA_NR1 | 50 | 61.21 | 8 | 20 | 4,252 | 1,484 | 1.681 | 1.483 | 0.231 | 0.191 |
| BA_NR2 | 50 | 42.53 | 5 | 9 | 4,252 | 1,484 | 1.671 | 1.476 | 0.228 | 0.182 |
| BA_NR3 | 49 | 51.39 | 7 | 24 | 4,252 | 1,484 | 1.689 | 1.5 | 0.23 | 0.189 |
| BA_NR4 | 49 | 49.81 | 6 | 19 | 4,252 | 1,484 | 1.681 | 1.496 | 0.23 | 0.186 |
| BA_NR5 | 50 | 43.04 | 6 | 14 | 4,252 | 1,484 | 1.711 | 1.519 | 0.234 | 0.193 |
| BA_NR6 | 50 | 45.56 | 7 | 19 | 4,252 | 1,484 | 1.664 | 1.477 | 0.227 | 0.18 |
| BA_NR7 | 50 | 85.38 | 4 | 9 | 4,252 | 1,484 | 1.685 | 1.491 | 0.233 | 0.191 |
| BA_NR8 | 48 | 79.48 | 6 | 18 | 4,252 | 1,484 | 1.675 | 1.471 | 0.229 | 0.183 |
| BA_NR9 | 50 | 37.03 | 5 | 14 | 4,252 | 1,484 | 1.664 | 1.501 | 0.229 | 0.186 |
| BA_SR1 | 50 | 64.00 | 5 | 28 | 4,252 | 1,484 | 1.665 | 1.637 | 0.229 | 0.243 |
| BA_SR2 | 50 | 27.09 | 4 | 7 | 4,252 | 1,484 | 1.665 | 1.66 | 0.228 | 0.258 |
| BA_SR3 | 50 | 18.60 | 9 | 32 | 4,252 | 1,484 | 1.686 | 1.67 | 0.233 | 0.261 |
| BA_SR4 | 50 | 41.81 | 8 | 14 | 4,252 | 1,484 | 1.678 | 1.659 | 0.231 | 0.256 |
| BA_SR5 | 50 | 51.95 | 2 | 7 | 4,252 | 1,484 | 1.697 | 1.679 | 0.233 | 0.26 |
| BA_SR6 | 50 | 63.74 | 5 | 12 | 4,252 | 1,484 | 1.651 | 1.615 | 0.224 | 0.236 |
| BA_SR7 | 50 | 70.97 | 5 | 12 | 4,252 | 1,484 | 1.677 | 1.693 | 0.229 | 0.27 |
| SP_RA1 | 46 | 64.42 | 20 | _ | 4,927 | 1,053 | 1.733 | 1.549 | 0.258 | 0.196 |
| SP_RA2 | 50 | 100.00 | 21 | _ | 4,927 | 1,053 | 1.735 | 1.568 | 0.26 | 0.211 |
| SP_SE1 | 30 | 49.05 | 13 | _ | 4,927 | 1,053 | 1.771 | 1.806 | 0.262 | 0.317 |
| SP_SE2 | 49 | 27.52 | 6 | _ | 4,927 | 1,053 | 1.729 | 1.741 | 0.255 | 0.307 |
| SP_SE3 | 45 | 16.55 | 9 | _ | 4,927 | 1,053 | 1.726 | 1.666 | 0.257 | 0.252 |
| SP_SE4 | 47 | 19.55 | 8 | _ | 4,927 | 1,053 | 1.771 | 1.764 | 0.264 | 0.313 |
|  | **48.3**  **(±4.34** SD**)** | _ | _ | _ | _ | _ | **1.696 (±0.03)** | **1.596 (±0.11)** | **0.238 (±0.01)** | **0.230 (±0.05)** |

Nº = Number of individuals collected; FC = Percentage of forest in the landscape; RI = Richness of birds that defecate or regurgitate *Euterpe edulis* seeds; AB = Abundance of birds that defecate or regurgitate *Euterpe edulis* seeds; N-SNPs = Number of biallelic neutral SNPs; O-SNPs = Number of biallelic outlier SNPs ; N-Alleles = Average number of alleles per neutral locus; O-Alleles = Average number of alleles per outlier locus; N-H_E_ = Neutral genetic diversity; O-H_E_ = Outlier genetic diversity; SD = Standard deviation.

.


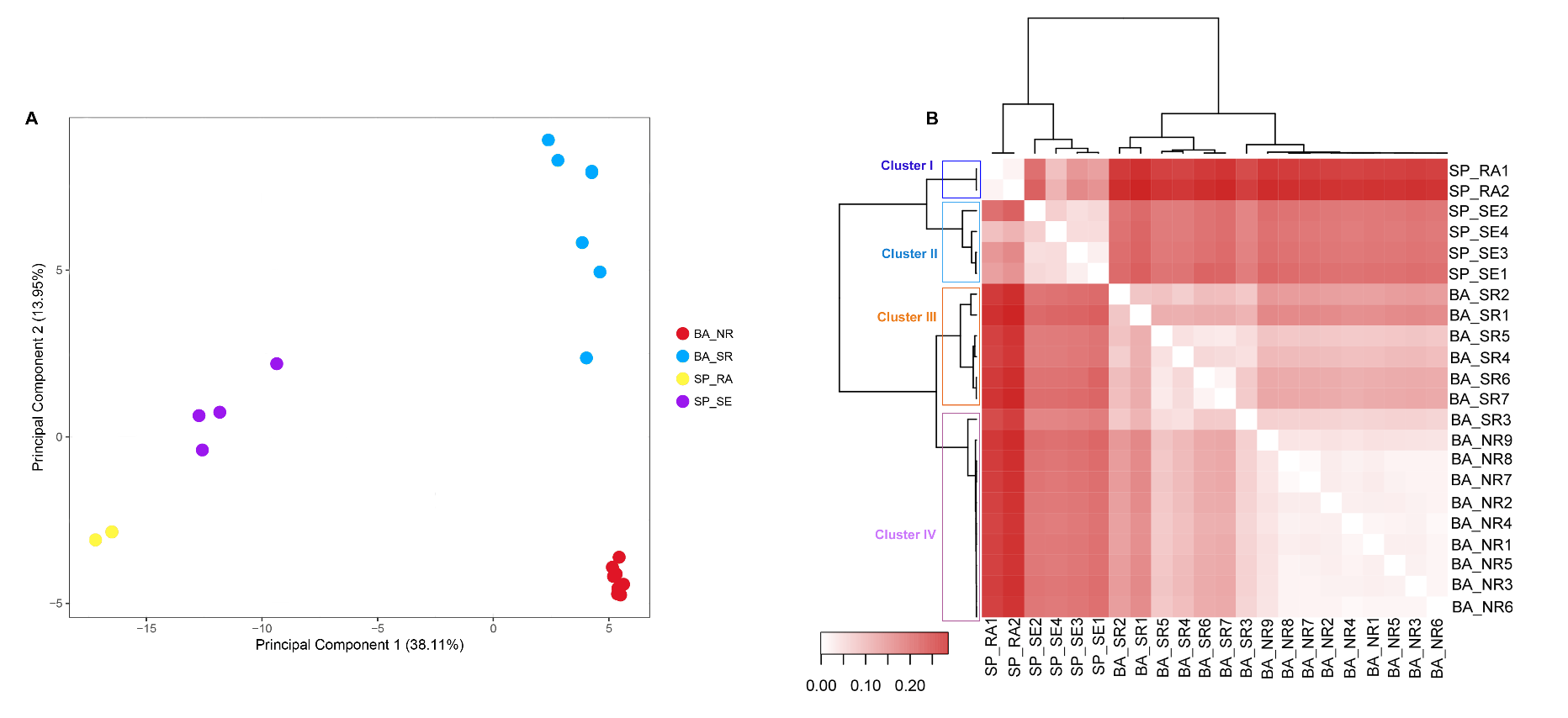


**Figure S1.** Characterization of the global genetic structure: (A) Genetic distribution of the 22 populations of *Euterpe*  *edulis* based on the allele frequencies of the SNPs according to Principal Component Analysis (PCA) and (B) Heatmap of the 22 populations of *E. edulis* based on Euclidean distance and the average clustering method using F_ST_ values. The four groups established (Cluster I, lilac rectangle; Cluster II, blue rectangle; Cluster III, orange rectangle; Cluster IV, pink rectangle), cluster 1 = SP_RA, cluster 2 = SP_SE, cluster 3 = BA_SR and cluster 4 = BA_NR).

**
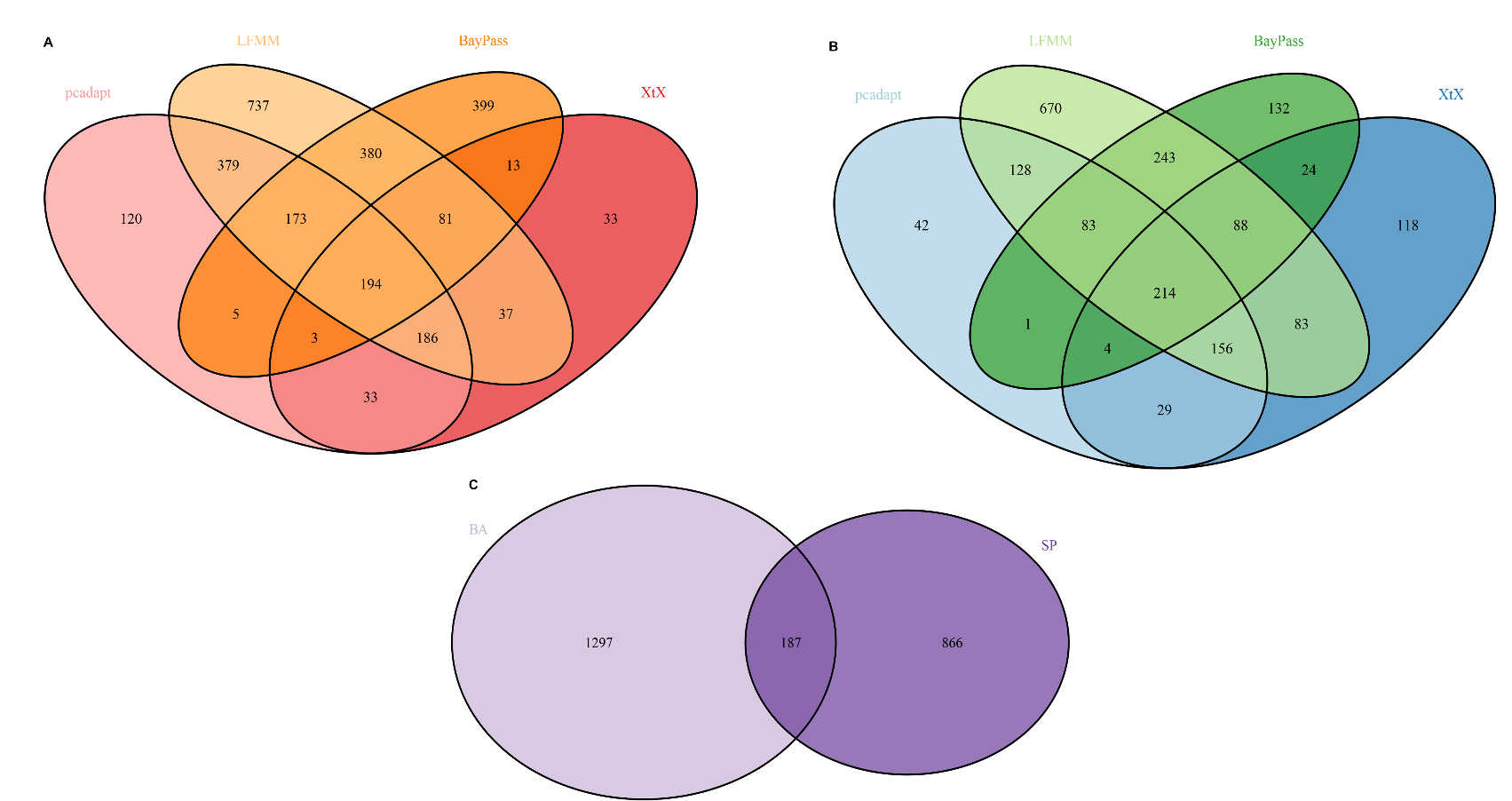
**

**Figure S2.** Venn diagram for SNPs detected as outliers (putatively under selection) using pcadapt, LFMM, BayPass and XtX in the 16 populations of *E. edulis* in BA (A); in the six populations of SP (B) and; the overlapping of SNPs identified as outlier by two or more tests simultaneously in BA and SP (C).


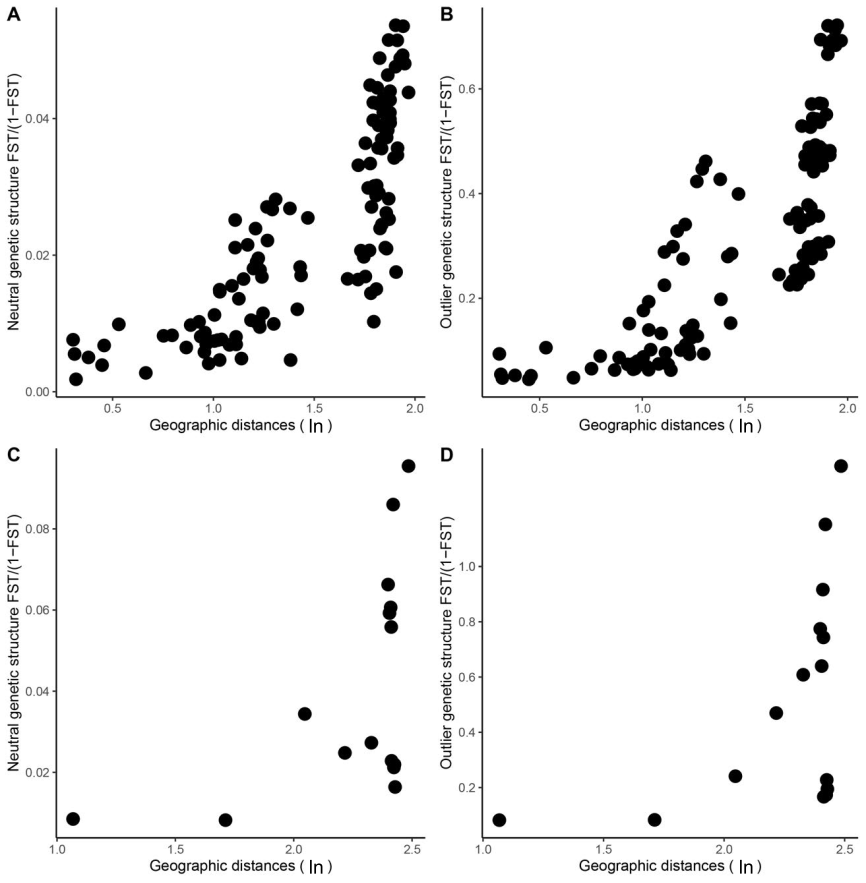


**Figure S3.** Relationships between F_ST_/(1-F_ST_) and log geographic distances in *E. edulis* populations within each state: (A) neutral structure and (B) outlier in BA; (C) for the neutral structure and (D) outlier in SP. Note that the transformation did not produce a linear relationship between F_ST_/(1-F_ST_) and log geographic distances, demonstrating that the process of isolation by geographic distance does not apply to the populations under study (Rousset,1997).
